# Working Conditions of Employee Optometrists in Australia

**DOI:** 10.1007/s44402-026-00050-2

**Published:** 2026-03-25

**Authors:** Rene Cheung, Matt Trinh, Vincent Lim, Rory Dowdall, Nicola Anstice

**Affiliations:** 1 School of Optometry and Vision Science, University of New South Wales, Sydney, Australia; 2 Centre for Eye Health, University of New South Wales, Sydney, Australia; 3 College of Nursing and Health Sciences, Flinders University, Bedford Park, Australia

**Keywords:** Job satisfaction, Optometry, Primary health care, Surveys and questionnaires, Value-based health care

## Abstract

**Purpose:**

Improving job satisfaction can mitigate workplace burnout, which refers to feelings of exhaustion and reduced professional efficacy, and preserve service quality. Understanding factors that improve job satisfaction is important, as a high proportion of Australian optometrists report burnout and conflicting pressures to meet financial benchmarks in the clinic. This study investigated factors impacting job satisfaction by assessing the demands placed on optometrists and resources available to manage workplace stressors.

**Methods:**

Optometrists were invited to complete an online survey probing demographic, employment, workplace and appointment book management characteristics. Their impact on job satisfaction was assessed using items from the job demands-resources model. Job satisfaction was elicited by the key outcome statements ‘*I have satisfactory career options and professional growth*’, ‘*I am satisfied with my income’* and ‘*I am satisfied with my current scope of practice and level of autonomy’*. The Clinician Experience Measure (CEM) was also administered for baseline purposes.

**Results:**

In total, 370 responses with a >50% completion rate were received, representing 5% of AHPRA-registered optometrists. The mean (SD) age was 36.1 (10.8) years, and 61.1% were female. Participants were least satisfied with career options and professional growth (22.5%) and income (24.6%), while only 43.0% were satisfied with their practice scope and level of autonomy. The lowest-scoring items on the CEM pertained to limited involvement in workplace decision-making. Expecting stable employment within the next year (*p* = 0.004–0.01) and longer appointment waiting times (*p* < 0.001–0.01) increased job satisfaction across all outcomes, while difficulty seeing emergency patients reduced satisfaction (*p* < 0.001). Income (*p* < 0.001), work setting (*p* < 0.001–0.02) and follow-up appointment times (*p* = 0.006) were also significant factors.

**Conclusions:**

Appointment schedules that allow for adequate follow-up time and can easily accommodate emergency consultations are the strongest modifiable factors available to employee optometrists for reducing workplace stressors. These findings can inform strategies for improving job satisfaction to optimise eyecare delivery.

Key Points

- Approximately a quarter of optometrists were not satisfied with their income, career options and opportunities for professional growth.
- Modifiable factors that can increase job satisfaction include longer follow-up appointment times and increased capacity to see emergency patients, which can reduce burnout in optometry and improve the quality of services.
- Job stability, longer patient waiting times for appointment bookings, income and work setting also influence job satisfaction among employee optometrists.

## Introduction

Australian optometrists play a crucial role in delivering primary eyecare services, including refractive correction, paediatric vision assessments, ocular disease screening and management and low vision rehabilitation [1, 2]. Job satisfaction, defined as the feelings that connect workers to their job and the extent to which workers like or dislike their work [3, 4], influences the quality of services provided [5]. Conversely, burnout refers to feelings of exhaustion, negativity and reduced efficacy in a workplace context, which negatively impacts job satisfaction and is experienced by 23.1–57.1% of Australian optometrists according to a survey conducted in 2021 [6]. Excessive workload and retail pressures to meet financial performance indicators due to short consultation times and insufficient breaks are often cited as key issues contributing to burnout [6, 7], which is associated with malpractice in other health professionals, including nurses [8] and physicians [9]. Failing to address these problems reduces job satisfaction and consequently could increase workforce attrition and reduce the availability of eyecare services, particularly outside metropolitan areas [10]. Indeed, an analysis of optometrists registered with the Australian Practitioner Regulation Agency within the last 5 years in 2023 found that the replacement rate declined in 2022 and only 79% intended to continue in the profession [11]. Recommendations have so far focused on improving the mental resilience of optometrists and lifestyle modifications to cope with workplace stressors [6, 12, 13]. Thus, further work is needed to identify both workplace and clinician factors that can increase job satisfaction and mitigate the effects of burnout, enabling optometrists to provide high-quality eyecare.

**Fig. 1:**
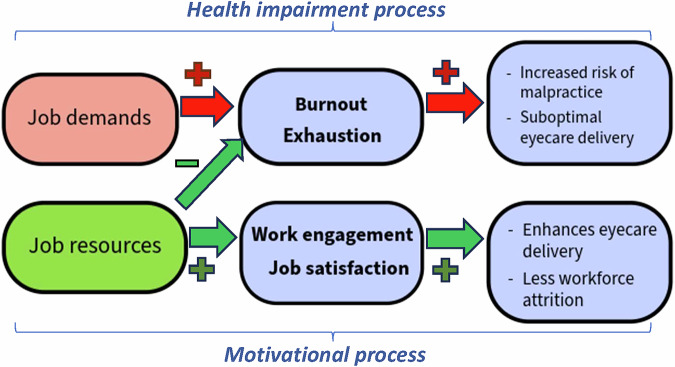
Flow chart of the job demands-resources model for assessing job satisfaction and burnout among employee optometrists. Adapted from Schaufeli et al. [17]. Arrows represent contributory relationships, e.g., job demands on burnout symptoms (top row) and job resources on work engagement/satisfaction (bottom row). Plus and minus annotations indicate the direction of impact between demands and resources on burnout, worker engagement and job satisfaction, and consequent outcomes.

The job demands-resources (JD-R) Model (Fig. 1) is a framework used to investigate workplace resources, demands and employee characteristics that influence work engagement, exhaustion, burnout [14, 15] and job satisfaction. It proposes that too many demands and insufficient resources at work can negatively impact employee job satisfaction and increase burnout [16, 17]. Job demands refer to workload, time pressures, administrative burden, emotional and cognitive demands, whereas resources include professional autonomy, collegial/management support and professional development opportunities [18].

The aim of this study was to investigate job satisfaction by assessing job demands and resources to cope with workplace stressors among employee optometrists in Australia using a cross-sectional survey design. The findings are significant for individual optometrists and professional organisations interested in identifying factors to reduce burnout and improve job satisfaction among employees and consequently, the quality of eyecare services.

## Methods

Researchers at Flinders University and the University of New South Wales co-designed the questionnaire with industry stakeholders—Optometry Australia (optometry.org.au/) and a recently established advocacy group, Phoropter Free Fridays [19]. The research protocol was approved by the Flinders University Human Research Ethics Advisory Committee (HREC 7859; December 2024). Only employed optometrists currently holding general registration with the Optometry Board of Australia and practising in a full-time, part-time, casual or locum capacity were invited to continue participating in the online survey as part of the inclusion criteria.

### Questionnaire Design

#### Demographic and Practice Characteristics

Basic demographic and employment characteristics were elicited by asking participants for their age, gender identity, annual income (before tax and excluding superannuation), year of entry into the Australian optometry workforce and number of practice locations they were currently employed at. At their main workplace, participants were also probed on length of employment, type of practice they work in (e.g., independent, corporate or ophthalmology practice), hours of practice per week, weeks worked per year, average number of patient examinations conducted per week, time allocated to initial comprehensive and follow-up eye examinations, administrative tasks and breaks in their appointment book. The capacity to provide walk-in and emergency appointments, average wait time for routine patient bookings, whether this had changed within the last 12 months or was expected to change in the next 12 months, were also elicited. Lastly, optometrists were asked about their expectations for future hours of employment and how their workplace was funded (see Table S1 for questionnaire items).

#### Job Satisfaction Measures

In accordance with previous recommendations on applying the JD-R model, items from the Energy Compass questionnaire were adapted to assess factors influencing job satisfaction, which includes a broad set of valid and reliable indicators while minimising response burden [17, 20]. Item selection was guided by extensive feedback collected from 102 and 140 Australian optometrists by Optometry Australia and Phoropter Free Fridays surveys (unpublished data), respectively, on demands experienced and resources available to employee optometrists:

- *Demands*: the qualitative demands assessed included feelings of professional isolation (emotional), geographical proximity of workplace (physical) and job security (work–home conflict). Quantitative (workload) and organisational demands, such as bureaucratic factors, i.e., annual leave/work hour flexibility and staff turnover, were also evaluated [17].
- *Resources*: social job resources assessed included role clarity, co-worker support and recognition of achievements by management. Work resources, including the ability to access administrative support, participate in collaborative care, the extent of staff support and the ability to exercise scope of practice were also assessed. Organisational (satisfaction with income) and developmental resources (opportunities for professional education and career growth) were probed (Table 1) [17].

**Table 1:** Items from the Energy Compass questionnaire mapped to factors in the JD-R model [17].

Job demands	Questionnaire item
Qualitative	
Emotional	I feel professionally isolated
Physical	I am satisfied with the geographical location where I am employed
Work–home conflict *(fear of unemployment or occupational career problems)*	I have good job security
Quantitative	
Workload	On most days, my workload is reasonable
Organisational	
Bureaucracy	I can schedule annual leave when I would like to have it
	Flexible work hours are accommodated at my practice
Negative change	There is a high level of staff turnover at the practice where I work
**Job resources**	**Questionnaire item**
Social	
Role clarity	My manager understands my role as an optometrist
Co-worker support	I get along well with my colleagues
Recognition	My manager values the work that I do
Work	
Job control	I can access appropriate administrative support when I need it
Participation in decision-making	I am able to actively participate in collaborative care for managing ocular diseases
Staff support	My practice has sufficient staff with the right mix of skills
Use of skills	I am satisfied with my current scope of practice and level of autonomy
Organisational	
Fair pay	I am satisfied with my income
Developmental resources	
Possibilities for learning and development	My work prioritises my ability to access continuing professional development (CPD) opportunities
	I have satisfactory career options and professional growth

*JD-R* job demands-resources.

A further ten items were adapted from the Clinician Experience Measure (CEM) questionnaire, a validated instrument that seeks to improve patients’ experience of receiving care and health outcomes [21], to complement the Energy Compass items by providing insight into clinicians’ experience of providing care. This establishes a benchmark that can be used to assess the impact of resource-demand modifications in a clinical context in the future. Items probing psychological safety, self-efficacy, interprofessional collaboration, quality of care and clinician engagement were included from the CEM (Table 2).

**Table 2:** Questionnaire items adapted from the Clinician Experience Measure (CEM) [21].

Clinician Experience Measure (CEM) factor	Item
Psychological safety	Members of staff in my practice are able to talk about problems and tough issues
	I feel safe to present new ideas and challenge current practice in my optometry practice
Self-efficacy	I am confident that I am able to provide high-quality patient care
Interprofessional collaboration	My colleagues and I make changes to our working approaches based on each other’s feedback
	My colleagues and I share decision-making power with each other
Quality of care	I am able to be responsive to the needs of individual patients to create a positive patient experience
	I am able to provide care aligned with the currently accepted best practice
Clinician engagement	I have the opportunity to participate in decision-making in my practice
	My voice is heard in the process of making a change in my practice
	My contributions are valued in decision-making in my practice

The draft instrument was pilot tested by three practising community optometrists. Minor modifications to items probing income and age were made to improve survey administration, e.g., using free response rather than multiple choice formats. The final survey was structured into four sections:

1. *Demography*: probed participant characteristics using multiple-choice questions.
2. *Workplace characteristics*: probed practice characteristics using a combination of multiple choice, multiple response and short answer questions.
3. *Job satisfaction*: contained the key questions on job satisfaction and evaluated job resources and demands in relation to optometry work and items on other factors using 5-point Likert scale items.
4. *Clinical Experience Measure* probed factors influencing optometrists’ experience of providing care in clinical practice using 5-point Likert scale items.

A free-response section was provided at the end of the survey for any additional comments (Table S5). The complete items in Sections 1 (demographic data) and 2 (workplace characteristics) are available in Table S1. Sections 3 and 4 applied 5-point Likert scales to capture agreement with statements, which were mapped to a score of 1 to 5: strongly disagree (score 1), disagree (score 2), neither agree nor disagree (score 3), agree (score 4), strongly agree (score 5). The mean of all item responses across participants were mapped to appropriate descriptive statements and rounded if necessary (e.g., a mean score of 3.5 out of 5 was mapped to overall agreement).

### Survey Administration

Employee optometrists holding general registration with the Australian Health Practitioner Regulation Agency (AHPRA) and who performed clinical work on a regular basis were invited to participate. The survey was advertised via Optometry Australia social media platforms and other affiliated Facebook and LinkedIn accounts over a 6-week period from December 2024 to January 2025. The approximate reach of this recruitment method is 85% of registered optometrists, given the national coverage of Optometry Australia membership [22]. The survey was administered via Qualtrics survey software (qualtrics.com). No financial incentives were offered for completing the survey.

### Sample Size Estimate

As of December 2024, there were 7405 optometrists registered with AHPRA in Australia [10]. Thus, 366 responses were required to provide adequate representation of the profession with 95% confidence and a 5% margin of error.

### Statistical Analysis

Responses were recorded using Qualtrics software and exported into Microsoft Excel (microsoft.com) before being transposed to SPSS for data analysis. Categorical variables were described by frequencies, and continuous data, including Likert scale responses, mean and standard deviation (SD) [23]. Univariable and multivariable backward linear regression were used to explore relationships between independent variables in Sections 1 and 2 to key (primary outcome) questions probing job satisfaction: ‘*I have satisfactory career options and professional growth*’, ‘*I am satisfied with my income’* and ‘*I am satisfied with my current scope of practice and level of autonomy*’. Two-sided 5% significance levels were used to define statistical significance. Differences greater than 0.5 SD between Likert scales were accepted as clinically meaningful [24]. Scores of 1–3 and 4–5 were dichotomised to binary outcomes, disagreement and agreement, for further analysis. All statistical analyses were conducted using SPSS (Version 25; IBM, ibm.com).

### Ethics Approval

This study was approved by Flinders University Human Research Ethics Advisory Committee (HREC 7859; December 2024).

## Results

### Response Rate

In total, 505 survey responses were received, with complete responses (*n* = 370) capturing 5% of optometrists holding general registration in Australia, which likely represents a greater subset of employed optometrists. Of all responses, 74.3% (375/505) completed more than 50% of the questionnaire, and the completion rate of key questions ranged from 99.5 to 100%, with at least 80% of participants providing a response to all other items in the survey (85.8–100%). Response rates and thresholds for including data were determined using standard definitions established by the American Association for Public Opinion Research (AAPOR). That is, surveys missing more than 50% of responses (*n* = 130) and items missing more than 80% were excluded from analysis (*n* = 0) [25]. Four participants were excluded as they self-identified as employer optometrists in the free response comments. One participant was also excluded because they reported an annual income of AUD$800,000, which is more than six times the national median salary for optometrists in Australia [26].

### Participant Characteristics

#### Demographic Variables

The mean age (SD) of participants was 36.1 (11) years. In total, 61.1% (226/370) identified as female, 35.9% as male (132/370) and 1.9% (7/370) preferred not to say. On average, participants had 12.2 (11) years of experience in primary eyecare and earned AUD$108,105 (39,961) annually (unadjusted for work hours).

#### Employment Variables

On average, optometrists performed 31.4 (9) h of work and worked 4.2 (1.2) days per week. Most optometrists performed clinical work in at least 31 weeks each year (93.0%, 344/370) and expected their work hours to increase or remain the same in the next 12 months (66.5%, 246/370). The mean number of employment locations was 1.7 (1), and optometrists had been employed at their main place of work for 6.1 (6.7) years on average.

#### Workplace Variables

The most common main work setting reported was corporate or franchise practices (64.6%, 239/370), followed by independent practices (25.1%, 93/370), locum work (13.5%, 50/370) or academic or research institutions (6.5%, 24/370), while a small proportion worked in ophthalmology practices (4.1%, 15/370) and other hospital/healthcare clinics (3.2%, 12/370). Funding arrangements at practices were also identified by probing whether services were entirely covered by the national universal health insurance scheme, Medicare (‘bulk-billed’), private fees or a combination of both. In total, 54.6% reported that all patients seen at their main workplace were bulk-billed (202/370), 28.4% (105/370) used mixed-billing fee structures by service and 10.0% (37/370) by patient category, while 6.8% (25/370) charged all patients privately.

#### Appointment Book Variables

Time allocated to initial, follow-up, administration duties and breaks varied widely among participants. For initial appointments, allocations ranged from 10 to 90 min per patient, and the mean and median duration were 28.7 and 30 min, respectively. Nine optometrists (2.4%) reported they had 15 min or less for initial consultations. One participant reporting 1.5 min per consult was excluded from the regression analysis due to possible misinterpretation of the questionnaire item (i.e., recorded response in hours instead of minutes). Follow-up appointment times ranged from 0 to 100 min, with mean and median times of 21.5 and 20 min, respectively. Ten optometrists (2.7%) indicated that they receive no time allocation for follow-up appointments.

Administration and break times allocated per week were also elicited, which showed that 62.7% (232/370) of participants were not allocated time to perform non-patient-facing duties (median: 0 min). The median time allocated to breaks per week was 60 min, with most optometrists reporting they had 30 min (31.6%, 117/370), followed by 150 min (18.9%, 70/370). These values should be interpreted with caution, as employees are legally required to have at least a 30-min meal break if they work more than 5 h per shift in Australia [27], and two-thirds of participants worked more than 24.5 h per week. It is possible that some participants may have misinterpreted the questionnaire item and reported the break time allocated per day instead.

### Appointment Capacity and Wait Time

The ability to accommodate additional exams and same-day emergency and walk-in patients was also probed to understand potential pressures faced by employee optometrists in clinical practice. In total, 60.8% (225/370) could accommodate additional routine exams through the week. Approximately half of the participants reported that it was easy to provide emergency appointments (58.6%, 217/370). While 84.1% (311/370) could offer same-day appointments to walk-in patients, 59.2% (219/370) reported that they are overworked in doing so. Participants indicated there was good overall capacity to provide timely appointments, with 76.5% (283/370) of optometrists advising they could offer appointments within 3 days, and just over half indicating that there was no change to this capacity within the last 12 months (58.1%, 215/370). All participant characteristics are summarised in Table 3.

**Table 3:** Participant characteristics.

Demographic variables	Results
Age (years)	
Mean (SD)	36.1 (10.8)
Median (range)	32.0 (22–67)
Gender, % (*n*/*N*)	
Female	61.1 (226/370)
Male	35.9 (132/370)
Prefer not to say	1.9 (7/370)
No response	0.5 (2/370)
Clinical experience (years)	
Mean (SD)	12.2 (11.0)
Median (range)	8.0 (1–47)
Income ($AUD/annum)^a^	
Mean (SD)	108,105 (39,961)
Median (range)	108,000 (10,800–400,000)
Employment variables	
Hours worked/week	
Mean (SD)	31.4 (9.0)
Median (range)	36.3 (4.5–40)
Workdays/week	
Mean (SD)	4.2 (1.2)
Median (range)	4.8 (0.6–5.3)
Employed weeks/year, % (*n*/*N*)	
<10	0.8 (3/370)
11–20	0.5 (2/370)
21–30	0.5 (2/370)
31–40	5.1 (19/370)
41+	93.0 (344/370)
Expected hours within the next 12 months, % (*n*/*N*)	
Remain unchanged	55.4 (205/370)
Increase	11.1 (41/370)
Decrease	26.8 (99/370)
Plan to leave profession	6.2 (23/370)
Retire	0.3 (1/370)
No response	0.3 (1/370)
Number of employment locations^b^	
Mean (SD)	1.7 (1.0)
Median (range)	1.0 (1–5)
Employment duration (years)	
Mean (SD)	6.1 (6.7)
Median (range)	4.0 (0–40)
Workplace variables	
Main work setting (>1 allowed)	
Corporate/franchise	64.6 (239/370)
Independent/private practice	25.1 (93/370)
Locum	13.5 (50/370)
Academic/research institution	6.5 (24/370)
Ophthalmology practice	4.1 (15/370)
Other	3.2 (12/370)
Hospital/clinic/healthcare facility	3.2 (12/370)
Workplace funding, % (*n*/*N*)	
All patients bulk-billed	54.6 (202/370)
Mixed billing by service	28.4 (105/370)
Mixed billing by patient category	10.0 (37/370)
All patients charged privately	6.8 (25/370)
Appointment book variables	
Initial exam duration (min)^c^	
Mean (SD)	28.7 (9.1)
Median (range)	30 (10–90)
Follow-up exam duration (min)	
Mean (SD)	21.6 (9.4)
Median (range)	20 (0–100)
Administration time/day (min)	
Mean (SD)	7.4 (14.8)
Median (range)	0.0 (0–91.8)
Break time/day (min)	
Mean (SD)	26.1 (25.1)
Median (range)	23.9 (0–250)
Appointment capacity and wait time	
Ability to accommodate additional exams, % (*n*/*N*)	
Yes	60.8 (225/370)
No	39.2 (145/370)
Difficulty seeing emergency patients, % (*n*/*N*)	
Very easy	7.3 (27/370)
Easy	51.4 (190/370)
Difficult	34.9 (129/370)
Very difficult	6.5 (24/370)
Ability to see walk-in patients, % (*n*/*N*)	
Able to provide an appointment (not overworked)	24.9 (92/370)
Able to provide an appointment (I am overworked)	59.2 (219/370)
Unable to provide an appointment	15.4 (57/370)
No response	0.3 (1/370)
Appointment waiting time, % (*n*/*N*)	
No wait time	21.9 (81/370)
1 day	22.7 (84/370)
2 days	20.0 (74/370)
3 days	11.9 (44/370)
4–7 days	13.2 (49/370)
8–14 days	7.0 (26/370)
15–30	1.9 (7/370)
>30 days	1.1 (4/370)
No response	0.3 (1/370)
Wait time 12 months ago, % (*n*/*N*)	
Shorter	19.7 (73/370)
Longer	21.9 (81/370)
Unchanged	58.1 (215/370)

*SD* standard deviation.

^a^Before tax and excluding superannuation (unadjusted for full-time hours).

^b^3.7% of participants worked in five or more practices (input as five for this analysis).

^c^One participant recording 1.5 min as their initial consultation was excluded.

### Job Demands-Resources Questionnaire

**Fig. 2:**
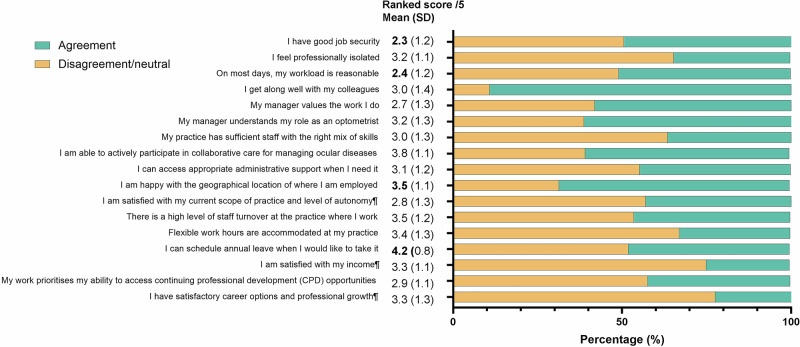
Dichotomised disagreement and agreement levels to each domain item in the JD-R model. ^¶^Key outcome measures. Strongly disagree, disagree and neither agree nor disagree were dichotomised to zero; agree and strongly agree to 1. Significant results (mean score rounding to either agreement or disagreement) are bolded. JD-R job demands-resources, SD standard deviation.

Employee optometrists reported overall job dissatisfaction with career options, professional growth and income, according to mean scores out of 5 (2.3 [1.3]–2.4 [1.2]) and the proportion of agreement (22.5–24.6%) measured in the questionnaire. Optometrists felt neutral about their scope of practice and clinical autonomy overall (mean score: 3.0 [1.3]), the third key job satisfaction outcome examined, with 43% indicating satisfaction on the dichotomised scale. After career options and professional growth or income, optometrists were least satisfied with the flexibility of work hours (2.7 [1.3], 32.7%) and the skill set of staff (2.8 [1.3], 36.5%), though this did not reach significance based on mean scores (Fig. 2).

Items to which participants showed clear agreement included satisfaction with workplace collegiality (4.2 [0.7], 89.2%), ability to actively participate in collaborative care of ocular diseases (3.5 [1.1], 60.3%), geographical location (3.8 [1.1], 68.2% agreement) and feeling that their role is understood by management (3.5 [1.2], 61.4%). The complete 5-point Likert scale responses to the JR-Demands model are shown in Table S2.

### Clinician Experience Measure

**Fig. 3:**
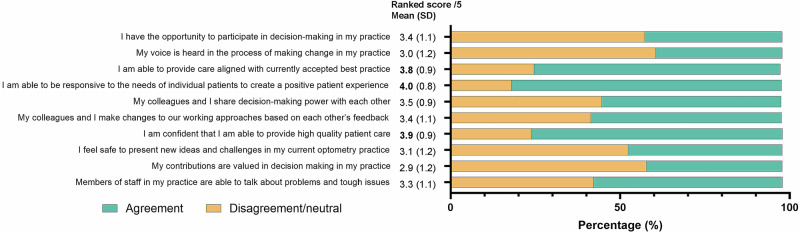
Dichotomised disagreement and agreement levels to each domain item in the Clinician Experience Measure. Strongly disagree, disagree and neither agree nor disagree were dichotomised to zero; agree and strongly agree to 1. Significant results (mean score rounding to either agreement or disagreement) are bolded. SD standard deviation.

Across the five dimensions of the CEM questionnaire, most participants agreed that they were able to provide quality patient care in clinical practice with significantly ranked mean scores (3.8 [0.9]–4.0 [0.8]; 72.7–79.7%). Participants felt neutral about items pertaining to psychological safety, self-efficacy, interprofessional collaboration and clinician engagement dimensions (2.9 [1.2]–3.4 [1.1]). The lowest-scoring items pertained to the degree that optometrists could participate in decision-making in the practice including ‘*My voice is heard in the process of making change in my practice*’ (2.9 [1.2], 37.3% agreement), ‘*My contributions are valued in decision making in my practice*’ (3.0 [1.2], 40% agreement), and ‘*I have the opportunity to participate in decision-making in my practice*’ (3.0 [1.2], 40.5% agreement; Fig. 3). However, none of these items reached significance based on mean scores. The complete 5-point Likert scale responses to the CEM are shown in Table S3.

### Factors Associated With Job Satisfaction

Univariable (Table S4) and multivariable linear regression analysis (Table 4) were conducted to identify factors influencing job satisfaction among employee optometrists. Unsurprisingly, higher earnings (*p* < 0.001) were associated with greater income satisfaction. For career and professional growth, greater clinical experience (*p* = 0.03) and mainly working at academic or research institutions (*p* = 0.02) showed positive, significant associations with satisfaction. In contrast, working mainly in corporate or franchise settings (*p* < 0.001) or as a locum optometrist (*p* = 0.002) significantly reduced satisfaction.

**Table 4:** Multivariable linear regression analysis of factors impacting primary job satisfaction outcomes.

	Satisfaction with income (*p*/*χ*^2^)	*β*	Satisfaction with career/professional growth (*p*/*χ*^2^)	*β*	I am satisfied with my current scope of practice and level of autonomy (*p*/*χ*^2^)	*β*
Demographic variables						
Age	0.60	−0.03	0.75	−0.05	0.62	−0.09
Income	**<0.001**	0.19	–	–	0.57	0.03
Experience (years)	–	–	**0.03**	0.11	0.18	0.07
Employment variables						
Expected hours within the next 12 months						
*Remain unchanged*	**0.01**	0.14	**0.01**	0.14	**0.004**	0.15
*Increase*	0.39	−0.05	–	–	–	–
*Decrease*	–	–	0.64	−0.04	0.76	−0.03
*Plan to leave profession or retire*	–	–	–		0.46	−0.04
Employment duration (years)	–	–	0.53	−0.04	–	–
Workdays/week	–	–	–	–	–	–
Workplace variables						
Main work setting (>1 allowed)						
*Corporate/Franchise*	0.11	−0.09	**<0.001**	−0.23	**<0.001**	−0.21
*Independent/Private practice*	0.51	−0.05	0.17	0.10	0.19	0.09
*Locum*	–	–	**0.002**	−0.18	0.06	−0.11
*Academic/research institution*	0.38	0.05	**0.02**	0.12	**0.03**	−0.12
*Hospital/clinic/healthcare facility*	–	–	–	–	–	–
*Ophthalmologist*	–	–	0.20	0.07	–	–
*Other*	–	–	–	–	–	–
Workplace funding						
*All patients bulk-billed*	0.69	−0.03	0.06	−0.16	0.84	0.01
*All patients charged privately*	–	–	0.43	0.05	0.23	0.06
*Mixed billing by patient category*	0.45	0.05	0.37	0.06	0.61	0.03
*Mixed billing by service*	–	–	0.05	0.10	–	–
Appointment book variables						
Initial exam duration (min)	0.43	−0.05	0.27	−0.07	0.49	−0.04
Follow-up exam duration (min)	0.05	0.11	0.69	0.02	**0.006**	0.15
Administration duration (min/day)	0.09	0.09	0.33	0.06	0.38	−0.05
Break duration (min/day)	–	–	0.12	0.08	–	–
Ability to accommodate additional exams (*reference: yes*)	0.30	−0.06	0.85	−0.01	0.11	−0.10
Difficulty seeing emergency patients	**<0.001**	−0.21	**<0.0001**	−0.22	**<0.0001**	−0.26
Ability to see walk-in patients *(reference: can provide an appointment)*	0.44	0.04	0.86	−0.01	–	–
Appointment wait time (*reference: no wait time*)	**<0.001**	0.20	**0.03**	0.12	**0.002**	0.17

Significant results (*p* < 0.05) are bolded.

For satisfaction with the current scope of practice and level of autonomy, longer follow-up appointment times had a significant positive effect (*p* = 0.006), whereas working mainly in corporate or franchise settings significantly reduced satisfaction (*p* = 0.001). For all outcomes, expectation of stable employment hours in the next 12 months and longer patient waiting times for appointments contributed to greater satisfaction (*p* = 0.004–0.01), while difficulty seeing emergency patients was a significant detractor (*p* < 0.001).

## Discussion

This study investigated job satisfaction of employee optometrists in Australia using a cross-sectional survey and presents quantitative tools that can be used for longitudinal evaluation and benchmarking. The investigation is timely as identifying factors that can mitigate burnout, which is increasingly reported among optometrists practising in other countries, including the United States [28, 29], Spain [3] and the United Kingdom [30], is crucial for optimising service quality and complements qualitative discussions on strategies to manage workplace stress for employers and employees [30]. These findings indicate that less than half of employee optometrists are satisfied with their income, career and professional growth, current scope of practice and autonomy. Most also do not feel empowered to make workplace changes. Ensuring that optometrists are allocated adequate time for follow-up appointments, have the capacity to accommodate emergency cases, and promoting work environments where optometrists can guide how they practice can potentially improve these outcomes. These are actionable strategies for reducing burnout, thereby improving job satisfaction so that patients receive the best care possible and support the ‘patient first’ approach outlined in the national Code of Conduct principles for health practitioners in Australia [31]. The present results parallel findings from the 2024 GOC Registrant Workforce and Perceptions Survey [32], which also reported that limited career progression and poor salaries contribute to job dissatisfaction among optometrists, with short testing times and reduced capacity for additional appointments perceived as negative influences on providing care.

### Factors Impacting Job Satisfaction

The survey found that dissatisfaction with career options, professional growth and income are potential drivers of burnout among employee optometrists, while good relationships with work colleagues, actively collaborating with other health professionals to manage ocular disease and recognition as health clinicians significantly offset job demands. This suggests that employee optometrists value positive work environments where their responsibilities and professional expectations are primarily clinical in nature. The findings mirror a recent survey investigating factors contributing to workforce attrition, which found that aside from retirement, lack of mental stimulation, unsatisfactory remuneration and lack of career advancement opportunities were the top reasons for leaving or intending to leave the optometric profession. Other previous workforce surveys have recommended addressing burnout by helping employees build resilience through mental health education during undergraduate training, workplace programmes [6, 12, 13] and lifestyle modifications to offset work-related stress [28]. While these are important strategies, the emphasis has been on individuals to cope with work stressors (job crafting) without exploring system-level factors (job redesign) that could potentially reduce burnout and job dissatisfaction.

### Modifiable

Greater follow-up times, capacity to see emergency patients and appointment waiting times improved job satisfaction outcomes. The median follow-up time reported was 20 min; however, 2.7% of optometrists indicated that they are allocated no time for follow-up appointments and essentially expected to ‘squeeze in’ these patients when necessary. Almost two-thirds of participants felt overworked when seeing same-day bookings, and 40% had difficulty fitting in emergency patients. Nine optometrists reported having less than 15 min for initial consultations, which falls below the minimum assessment times required for billing in the national public health system schedule (Medicare) [33]. These findings suggest that there is a lack of flexibility in appointment book schedules, leaving optometrists vulnerable to overwork when demand for services is high, which is potentially compensated for by reducing consultation times.

Greater flexibility in appointment schedules and longer appointment times have both been shown to reduce stress among medical and allied health clinicians [34, 35], which could alleviate job demands and optimise eyecare service delivery. Qualitative work by Retallic et al. suggested that allocating time to administrative duties and protecting breaks could reduce workplace stress [30]; however, break or administration time did not significantly influence job satisfaction in the present cohort. It is possible that there are no further benefits to job satisfaction after some threshold, particularly as administrative workloads can fluctuate.

While employee optometrists share the responsibility of managing appointment schedules, results from the CEM questionnaire suggest that a high proportion of employee optometrists do not feel empowered to make decisions at work, with nearly 40% reporting that they do not participate in decision-making and only 45% feeling comfortable with presenting new ideas. Thus, it is also important for practice managers and organisations to work collaboratively with employees and encourage them to raise issues that affect their work environment and clinical performance. Employers can also proactively work towards optimising clinical efficiency and managing workloads appropriately for employees to reduce the risk of burnout. For example, conducting regular diary reviews, managing late patients appropriately to minimise clinical workflow disruptions and adjusting appointment durations according to age or services required [30]. These initiatives can complement previously suggested strategies to encourage healthy lifestyles [28, 36], develop assertiveness among optometry students during university training and workers through further mental health education [6] to reduce burnout.

### Non-modifiable

Factors that are relatively non-modifiable from an employee and employer perspective include the type of work setting and how workplaces are funded. The direct relationship between career and professional growth satisfaction and years of clinical experience is reflective of survivorship bias [37], that is, those who continue to practice optometry over time are likely to be more satisfied with their overall career trajectory.

Greater satisfaction with career and professional growth, scope of practice and clinical autonomy amongst optometrists working in academic settings mirrors findings from a recent study in the United States, showing that optometrists working in academia have the lowest level of burnout [28]. Conversely, optometrists working in corporate or franchise practices were significantly less satisfied with their career and scope of practice. Indeed, 41.9% (155/370) of participants left free response comments at the end of the survey, with 29.7% of these criticising corporate practice environments for workplace dissatisfaction. The conflict of interest optometrists experience when faced with retail pressures in corporate work environments and excessive workloads has been cited previously in the literature and likely explains this association [6, 38], as shown by the following examples of feedback (see Table S5 for free response results) from corporate practice employees:

Comment 25: ‘*Targets are increasingly unrealistic and optometry KPI’s [key performance indicators] can be very demoralising for many of my colleagues who work very hard but don’t measure up to corporate optometry KPI’s*’

Comment 139: ‘*Its normal to not take a full 30 minute lunch break or quick toilet break due to full appointment books. Sometimes, we skip lunch altogether and ‘forget to eat’ to meet the demand of busy stores*.’

Optometrists who primarily practice as locums were also significantly less satisfied with their career and professional growth. This subgroup was employed in 2.7 locations on average compared to 1.7 for other employee types, worked significantly less hours (27.3 vs. 32.4 h), yet earned a similar income (AUD$107,052 vs. AUD$108,105, unadjusted for hours of work). There are two possible explanations for this association: (1) less satisfied optometrists may have chosen locum work for higher hourly remuneration with fewer hours, and (2) locum work across multiple sites may limit control over key determinants of job satisfaction, such as appointment book variables. The strong association between job stability and work satisfaction across all outcomes also indicates the importance of job security to employee optometrists.

Finally, most (78%) optometrists were dissatisfied with their current income. With 54.6% of workplaces limiting funding models to Medicare service rebates, there is little financial incentive from employers to increase remuneration, given that rebates have been indexed inadequately compared to inflation over the past 10 years [39, 40]. The limited scope of services covered by the schedule relative to services recommended in best practice guidelines [41–44] may also leave optometrists feeling undervalued, given that their scope of practice has expanded considerably in recent years to include therapeutic prescribing [1], glaucoma management [45, 46] and myopia control [47].

### Generalisability to Profession

The demographic profile of the present cohort was younger (36.1 vs. 41.6 years) and had less clinical experience (12.2 vs. 16.7 years) compared to workforce statistics reported by AHPRA [48] and the Australian Government Department of Health [49] in 2023 and 2019. This is expected given that only employee optometrists were invited to participate in the study, who are more likely to be early career with less financial and/or professional capacity to own or gain a partnership in a practice that employs other optometrists. Other demographic characteristics were similar to the broader profession, including gender identity (61.1 vs. 59.7% female [50]), salary (AUD$108,105 vs. AUD$110–120,000 [51]) and hours worked per week (31.4 vs. 34.6 h [49]). Interestingly, two-thirds of optometrists nominated corporate or franchise practices as their main workplace, whereas other recent national surveys were primarily completed by optometrists working in independent practices, i.e., only a third worked in corporate or franchise practices [52, 53]. Although there may be an element of response bias from employees unhappy with their workplace conditions, the most recent national statistics available on practice settings show that 62.5% of optometrists work in group private practices, suggesting these results are representative of the wider profession in this regard [49]. This could also explain the low percentage of optometrists charging private fees for all services compared to previous surveys [52, 53], given that major corporate group practices frequently advertise ‘no gap’ eyecare consultations [54–56].

### Limitations

The relatively small sample size of this study compared to other workforce surveys was a limitation, as previous response rates of 8.8–23% [6, 11, 57] among AHPRA-registered optometrists in Australia have been achieved. However, the potential sample was smaller as only employee optometrists were eligible to participate, and thus, the actual response rate in the target sample is underestimated. Other factors that could have affected response rates include conducting recruitment during the December to January period, when several key national public holidays occur and the use of stringent AAPOR criteria to determine whether a response was included. For example, if the initial 505 responses to the survey were included in the calculation, then the response rate increases to 6.8%. Nevertheless, the demographic profile of the cohort was generally similar to previous national reports. The results also cannot be generalised to employer optometrists (i.e., business owners). However, restricting participation to employees means that the results can directly inform strategies for optimising worker satisfaction at an organisational level, since other factors such as financial incentives or pressure could influence responses from employer optometrists. Potential misinterpretation of questionnaire items probing time allocations to administration and breaks at work also confounded the analysis. Future work should invest in further pilot testing of these items to refine the survey.

## Conclusion

Over 50% of employee optometrists in Australia are dissatisfied with their income, career and professional growth. The findings can assist organisations and practices that employ optometrists in reducing job dissatisfaction by creating work environments where employees feel involved in practice decision-making, have adequate time for follow-up appointments and emergency appointments. Regular discussions about remuneration and job stability can also have a significant impact on job satisfaction and optimise the quality of eyecare delivered to patients.

## Supplementary Information

Supplementary

## Data Availability

Available upon request.
