# Supplementary material for: Working Conditions of Employee Optometrists in Australia
Source: Ophthalmic Physiol Opt. 2026 Mar 25;46(2):285–95. doi: 10.1007/s44402-026-00050-2 (PMC13369529; doi:10.1007/s44402-026-00050-2)
Supplement: Supplementary file 1 — Supplementary [file 44402_2026_50_MOESM1_ESM.docx]

# Supplementary

### **Table S1**: Questionnaire items

**Section 1**

| ***Demographic data*** | ***Response options*** |
| --- | --- |
| Age  1.1 What is your age (in years) | ___________________________ |
| Gender  1.2 How do you describe your gender? (select one) | Male  Female  Non-binary / third gender  Prefer not to say |
| Income  1.3 What is your annual income (before tax and excluding superannuation or bonuses)? | ___________________________ |
| ***Workforce characteristics*** |  |
| Clinical experience  2.1 What year did you start practising optometry in Australia? | ___________________________ |
| Employment  2.2 How many hours, on average, are you scheduled to work per week? (select one) | 1-8 hours  8.5-16 hours  16.5-24 hours  24.5-32 hours  32.5-40 hours  >40 hours |
| 2.3 In the next 12 months, would you like the number of hours per week in patient care to: | Increase  Decrease  Remain unchanged  Plan to retire within the next year  Plan to leave the profession within the next year |
| 2.4 On average, how many weeks (excluding annual leave) do you work per year? (select one) | <10 weeks  11-20 weeks  21-30 weeks  21-40 weeks  41-50 weeks  51-52 weeks |
| 2.5 How many different practice locations do you work at, per week? (select one) | 1  2  3  4  5 or more |
| 2.6 Considering your primary employment, how long have you been in your current position (in years)? | ___________________________ |
| ***Workplace characteristics*** |  |
| 3.1 What is/are your main work setting(s)?  If you are employed by more than one practice type, select all that apply. | Employed by corporate/franchise (e.g. OPSM, Specsavers, Bupa, George &Matilda)  Employed by independent/private practice  Employed by hospital/clinic/health care facility  Employed by ophthalmologist  Employed by an academic or research institution  Employed as a locum  Other (please specify: ____________________) |
| 3.2 How is your primary workplace funded? | Medicare billing for all appointments  Private fees for all patient appointments  Private fees for most patients, and Medicare bulk billing for certain patient types e.g. seniors, children, students  Private fees for specific services and Medicare bulk billing for others |
| ***Appointment schedule characteristics*** |  |
| 4.1 On average, how many patient examinations (including initial and follow-up assessment) do you conduct per week? (select one) | 0-10 exams  11-20 exams  21-30 exams  31-40 exams  41-50 exams  51-60 exams  61-70 exams  71-80 exams  81-90 exams  >90 exams |
| 4.2 How long (in minutes) are you allocated for conducting an initial comprehensive eye examination (for example, an eye examination that you could bill a 10910 or 10911 to Medicare) in the appointment diary? | ___________________________ |
| 4.3 How long (in minutes) are you allocated for conducting follow-up appointments in the appointment diary? | ___________________________ |
| 4.4 In any given week, how long (in minutes) are you allocated for administration tasks in the appointment book? | ___________________________ |
|  |  |
| 4.5 In any given week, how long (in minutes) are you allocated for breaks (total breaks including tea and lunch breaks) in the appointment book? | ___________________________ |
| 4.6 In an average week, could you accommodate any additional patient examinations could you accommodate without changing hours, appointment allocation times or staffing? | I am fully booked and could not accommodate any additional patient exams  I could accommodate more patients |
| 4.7 How easy or difficult is it for you to see emergency patients at your primary workplace? | Very easy  Easy  Difficult  Very difficult |
| 4.8 In relation to patients requesting walk-in (no prior booking but wishing to be seen immediately) appointments, are you: | Unable to accommodate any patients requesting walk-in appointments  Able to provide care to patients who request walk-in appointments, but am overworked to accommodate this  Able to provide care to patients who request walk-in appointments and am NOT overworked to accommodate this |
| 4.9 How many days must a patient wait to obtain a routine appointment with you? | There is no waiting time, the patient can usually be seen the same day  1 day  2 days  3 days  4-7 days  8-14 days  15-30 days  >30 days |
| 4.10 Compared to 12 months ago, is the wait time: | Shorter  Longer  Unchanged |

**Section 2****: Job Satisfaction**

Select a response for each statement below to show how much you agree or disagree with it.

|  | Strongly disagree | Disagree | Neither agree nor disagree | Agree | Strongly Agree |
| --- | --- | --- | --- | --- | --- |
| I have satisfactory career options and professional growth |  |  |  |  |  |
| I am satisfied with my income |  |  |  |  |  |
| I am satisfied with my current scope of practise and level of autonomy |  |  |  |  |  |
| I am happy with the geographical location of where I am employed |  |  |  |  |  |
| I have good job security |  |  |  |  |  |
| I can schedule annual leave when I would like to take it |  |  |  |  |  |
| Flexible work hours are accommodated at my practice |  |  |  |  |  |
| My practice has sufficient staff with the right mix of skills |  |  |  |  |  |
| My manager understands my role as an optometrist |  |  |  |  |  |
| My manager values the work that I do |  |  |  |  |  |
| I get along well with my colleagues |  |  |  |  |  |
| I can access appropriate administrative support when I need it |  |  |  |  |  |
| On most days, my workload is reasonable |  |  |  |  |  |
| I am able to actively participate in collaborative care for managing ocular diseases |  |  |  |  |  |
| My work prioritises my ability to access continuing professional development (CPD) opportunities |  |  |  |  |  |
| I feel professionally isolated |  |  |  |  |  |
| There is a high level of staff turnover at the practice where I work |  |  |  |  |  |

**Section 3: Clinician Experience Measure Questionnaire**

Please indicate the extent to which you agree or disagree with the following statements in your role as an optometrist at your primary workplace:

|  | Strongly disagree | Disagree | Neither agree nor disagree | Agree | Strongly Agree |
| --- | --- | --- | --- | --- | --- |
| I am confident that I am able to provide high quality patientcare |  |  |  |  |  |
| I am able to be responsive to the needs of individual patients to create a positive patient experience |  |  |  |  |  |
| I am able to provide care aligned with currently accepted best practice |  |  |  |  |  |
| My colleagues and I make changes to our working approaches based on each others feedback |  |  |  |  |  |
| My colleagues and I share decision-making power with each other |  |  |  |  |  |
| Members of staff in my practice are able to talk about problems and tough issues |  |  |  |  |  |
| I feel safe to present new ideas and challenge current practice in my optometry practice |  |  |  |  |  |
| I have the opportunity to participate in decision-making in my practice |  |  |  |  |  |
| My voice is heard in the process of making change in my practice |  |  |  |  |  |
| My contributions are valued in decision making in my practice |  |  |  |  |  |

**Open ended comments**

Please provide any comments or additional insights that you would like to make, if any.

### **Table S2**: Complete participant responses to the Job-Demand Resources questionnaire

| **Domain/Item** | **Ranked score, mean (SD)** | **% (n/N) selected each category** | | | | |  |
| --- | --- | --- | --- | --- | --- | --- | --- |
|  |  | 1  Strongly disagree | 2  Disagree | 3  Neither agree nor disagree | 4  Agree | 5  Strongly agree | No response |
| ***Resources (Developmental)*** |  |  |  |  |  |  |  |
| I have satisfactory career options and professional growth* | 2.3 (1.2) | 34.1  (126/370) | 27.8 (103/370) | 15.7 (58/370) | 18.4  (68/370) | 4.1  (15/370) | 0  (0/370) |
| My work prioritises my ability to access continuing professional development (CPD) opportunities | 3.2 (1.1) | 8.4  (31/370) | 21.4 (79/370) | 27.8 (103/370) | 30.5  (113/370) | 11.6 (43/370) | 0.3  (1/370) |
| ***Resources (Organisational)*** |  |  |  |  |  |  |  |
| I am satisfied with my income* | 2.4 (1.2) | 27.0  (100/370) | 32.2 (119/370) | 15.7 (58/370) | 20.8  (77/370) | 3.8  (14/370) | 0.5  (2/370) |
| I can schedule annual leave when I would like to take it | 3.0 (1.4) | 17.8  (66/370) | 21.4 (79/370) | 12.7 (47/370) | 34.1  (126/370) | 13.5 (50/370) | 0.5  (2/370) |
| Flexible work hours are accommodated at my practice* | 2.7 (1.3) | 23.2  (86/370) | 24.9 (92/370) | 18.9 (70/370) | 22.4  (83/370) | 10.3  (38/370) | 0.3  (1/370) |
| There is a high level of staff turnover at the practice where I work | 3.2 (1.3) | 11.6  (43/370) | 23.0  (85/370) | 18.9  (70/370) | 26.2  (97/370) | 20.0  (74/370) | 0.3  (1/370) |
| ***Resources (Work)*** |  |  |  |  |  |  |  |
| I am satisfied with my current scope of practice and level of autonomy | 3.0 (1.3) | 15.4  (57/370) | 22.4 (83/370) | 19.2 (71/370) | 32.7  (121/370) | 10.3 (38/370) | 0  (0/370) |
| I am happy with the geographical location of where I am employed | 3.8 (1.1) | 5.1  (19/370) | 8.4  (31/370) | 17.8 (66/370) | 41.4  (153/370) | 26.8 (99/370) | 0.5  (2/370) |
| I can access appropriate administrative support when I need it | 3.1 (1.2) | 12.2  (45/370) | 20.3 (75/370) | 22.7 (84/370) | 34.3  (127/370) | 10.3  (38/370) | 0.3  (1/370) |
| I am able to actively participate in collaborative care for managing ocular diseases | 3.5 (1.1) | 3.5  (13/370) | 17.8  (66/370) | 17.8  (66/370) | 43.0  (159/370) | 17.3  (64/370) | 0.5  (2/370) |
| ***Resources (Social)*** |  |  |  |  |  |  |  |
| My practice has sufficient staff with the right mix of skills* | 2.8 (1.3) | 16.8  (62/370) | 31.6  (117/370) | 15.1  (56/370) | 27.6  (102/370) | 8.9  (33/370) | 0  (0/370) |
| My manager understands my role as an optometrist | 3.5 (1.2) | 11.1  (41/370) | 12.2  (45/370) | 15.4  (57/370) | 42.2  (156/370) | 19.2  (71/370) | 0  (0/370) |
| My manager values the work I do | 3.4 (1.3) | 10.5  (39/370) | 16.5  (61/370) | 14.9  (55/370) | 39.7  (147/370) | 18.4  (68/370) | 0  (0/370) |
| I get along well with my colleagues | 4.2 (0.8) | 0.5  (2/370) | 3.0  (11/370) | 7.3  (27/370) | 54.3  (201/370) | 34.9  (129/370) | 0  (0/370) |
| ***Demands (Quantitative)*** |  |  |  |  |  |  |  |
| On most days, my workload is reasonable | 3.3 (1.1) | 6.8  (25/370) | 21.1  (78/370) | 21.1  (78/370) | 42.2  (156/370) | 8.6  (32/370) | 0.3  (1/370) |
| ***Demands (Qualitative)*** |  |  |  |  |  |  |  |
| I feel professionally isolated* | 2.9 (1.1) | 7.8  (29/370) | 34.3  (127/370) | 23.2  (86/370) | 24.9  (92/370) | 9.5  (35/370) | 0.3  (1/370) |
| I have good job security | 3.3 (1.3) | 10.0  (37/370) | 22.2  (82/370) | 18.4  (68/370) | 32.2  (119/370) | 17.3  (64/370) | 0  (0/370) |

### **Table S3**: Complete participant responses to the Clinician Experience Measure questionnaire

| **Dimension** | **Ranked score, mean (SD)** | **% (n/N) selected each category** | | | | |  |
| --- | --- | --- | --- | --- | --- | --- | --- |
|  |  | 1  Strongly disagree | 2  Disagree | 3  Neither agree nor disagree | 4  Agree | 5  Strongly agree | No response |
| ***Psychological safety*** |  |  |  |  |  |  |  |
| Members of staff in my practice are able to talk about problems and tough issues | 3.4 (1.1) | 7.6  (28/370) | 16.2  (60/370) | 18.4  (68/370) | 46.8  (173/370) | 8.9  (33/370) | 2.2  (8/370) |
| My contributions are valued in decision making in my practice | 3.0 (1.2) | 13.5  (50/370) | 19.7  (73/370) | 24.6  (91/370) | 32.4  (120/370) | 7.6  (28/370) | 2.2  (8/370) |
| I feel safe to present new ideas and challenge current practice in my optometry practice | 3.1 (1.2) | 12.4  (46/370) | 18.9  (70/370) | 21.1  (78/370) | 37.3  (138/370) | 8.1  (30/370) | 2.2  (8/370) |
| ***Self-efficacy*** |  |  |  |  |  |  |  |
| I am confident that I am able to provide high quality patient care | 3.9 (0.9) | 1.4  (5/370) | 9.2  (34/370) | 13.2  (49/370) | 51.1  (189/370) | 23.0  (85/370) | 2.2  (8/370) |
| ***Interprofessional collaboration*** |  |  |  |  |  |  |  |
| My colleagues and I make changes to our working approaches based on each other’s feedback | 3.5 (0.9) | 1.9  (7/370) | 14.1  (52/370) | 25.4  (94/370) | 47.0  (174/370) | 9.2  (34/370) | 2.4  (9/370) |
| My colleagues and I share decision-making power with each other | 3.4 (1.1) | 5.4  (20/370) | 16.5  (61/370) | 22.7  (84/370) | 43.2  (160/370) | 9.7  (36/370) | 2.4  (9/370) |
| ***Quality of care*** |  |  |  |  |  |  |  |
| I am able to be responsive to the needs of individual patients to create a positive patient experience | 4.0 (0.8) | 1.1  (4/370) | 6.5  (24/370) | 10.3  (38/370) | 56.5  (209/370) | 23.2  (86/370) | 2.4  (9/370) |
| I am able to provide care aligned with currently accepted best practice | 3.8 (0.9) | 1.4  (5/370) | 8.9  (33/370) | 14.3  (53/370) | 52.7  (195/370) | 20.0  (74/370) | 2.7  (10/370) |
| ***Clinician engagement*** |  |  |  |  |  |  |  |
| My voice is heard in the process of making change in my practice | 2.9 (1.2) | 16.8  (62/370) | 23.2  (86/370) | 20.5  (76/370) | 29.5  (109/370) | 7.8  (29/370) | 2.2  (8/370) |
| I have the opportunity to participate in decision-making in my practice | 3.0 (1.2) | 14.6  (54/370) | 23.2  (86/370) | 19.5  (72/370) | 31.9  (118/370) | 8.6  (32/370) | 2.2  (8/370) |

### **Table S4:** Univariable linear regression analysis of associations between factors and primary outcome measures.

|  | **Satisfaction with income (p/χ^2^)** | **ᵦ** | **Satisfaction with career/ professional growth (p/χ^2^)** | **ᵦ** | **I am satisfied with my current scope of practice and level of autonomy** | **ᵦ** |
| --- | --- | --- | --- | --- | --- | --- |
| **Demographic variables** |  |  |  |  |  |  |
| Age | **0.04** | 0.12 | **0.002** | 0.17 | **0.002** | 0.17 |
| Income | **<0.0001** | 0.23 | 0.07 | 0.10 | **0.03** | 0.12 |
| Experience (years) | 0.08 | 0.10 | **0.001** | 0.19 | **0.002** | 0.17 |
| **Employment variables** |  |  |  |  |  |  |
| **Expected hours within next 12 months**  *Remain unchanged*  *Increase*  *Decrease*  *Plan to leave profession or retire* | **<0.001**  **0.04**  0.11  0.10 | 0.19  -0.11  -0.09  -0.09 | **<0.001**  0.64  **0.04**  **0.001** | 0.20  -0.02  -0.11  -0.17 | **<0.001**  0.49  **0.04**  **0.004** | 0.20  -0.04  -0.11  -0.15 |
| Employment duration (years) | 0.73 | 0.02 | **0.04** | 0.11 | **0.01** | 0.13 |
| Work days (/week) | 0.21 | -0.07 | 0.78 | -0.01 | 0.89 | -0.01 |
| **Workplace variables** |  |  |  |  |  |  |
| **Main work setting (>1 allowed)**  *Corporate/franchise*  *Independent/private practice*  *Locum*  *Academic/research institution*  *Hospital/clinic/health care facility*  *Ophthalmologist*  *Other* | **<0.0001**  **0.01**  0.18  **0.04**  0.33  0.14  0.92 | -0.21  0.14  -0.07  0.11  0.05  0.08  0.01 | **<0.0001**  **<0.0001**  **0.01**  **<0.001**  0.58  **0.004**  0.94 | -0.27  0.26  -0.14  0.19  0.03  0.15  0.004 | **<0.0001**  **<0.0001**  **0.04**  0.13  0.24  0.06  0.24 | -0.28  0.28  -0.11  0.08  0.06  0.10  -0.06 |
| **Workplace funding**  *All patients bulk-billed*  *All patients charged privately*  *Mixed billing by patient category*  *Mixed billing by service* | **0.001**  0.42  **0.01**  0.15 | -0.18  0.04  0.15  0.08 | **<0.0001**  **0.002**  **<0.001**  **0.02** | -0.32  0.16  0.20  0.13 | **<0.0001**  **<0.001**  **<0.001**  0.81 | -0.23  0.20  0.19  0.01 |
| **Appointment book variables** |  |  |  |  |  |  |
| Initial exam duration (min) | **0.01** | 0.15 | **<0.0001** | 0.21 | **<0.0001** | 0.28 |
| Follow-up exam duration (min) | **<0.001** | 0.19 | **0.001** | 0.18 | **<0.0001** | 0.27 |
| Administration duration (min, /day) | **0.002** | 0.17 | **<0.0001** | 0.21 | **0.05** | 0.10 |
| Break duration (min, /day) | 0.07 | 0.10 | **<0.001** | 0.18 | 0.14 | 0.08 |
| Ability to accommodate additional exams (*reference: yes*) | **0.02** | -0.13 | **0.001** | -0.18 | **<0.001** | -0.19 |
| Difficulty seeing emergency patients | **<0.0001** | -0.22 | **<0.0001** | -0.25 | **<0.0001** | -0.28 |
| Ability to see walk-in patients *(reference: can provide an appointment)* | **<0.0001** | 0.21 | **0.002** | 0.16 | 0.34 | 0.05 |
| Appointment wait time (*reference: no wait time*) | **<0.0001** | 0.21 | **<0.001** | 0.20 | **<0.0001** | 0.20 |

### **Table S5**: Open ended comments

| **Comment #** | | **Description** | | |
| --- | --- | --- | --- | --- |
| 1 | | - dispensing staff turn over is very high, they don’t get paid enough for have enough growth to continue their career  - I am provided no additional breaks other than an unpaid 1 hour lunch break. I have not taken this lunch break fully in the last 6 years (excluding covid period)  - despite asking for administrative time to do letters I was denied. I brought up the health practitioners act and OA advice about 15 minute breaks and was told that I could take that if I finished early with a patient which never happens as my patients are mostly 70 plus  - I often stay back after work to complete reports and referrals- I have kept a log and each month I and writing 80 letters on average - we need to specifically be told we are part of the health practitioner act and there needs to be an enforceable admin time and 2 15 minute breaks each day  - I think there should be a Medicare cap on number of patients seen per day (no more than 16)  - I think there needs to be heavy fines issued to any businesses who do not provide their employees with breaks or admin time as the work load is not reasonable  - there should be no requirement for reasonable over time which is used against us all the time. Doing more than 3 hours of overtime each week is crazy and is causing most optometrist to skip steps- which is dangerous! | | |
| 2 | | I find my work deeply meaningful and feel privileged that patients trust me with their eye and vision health needs. However, the challenge of balancing high-quality care with tight time constraints has taken a toll. Being chronically “productive” at work comes at a cost—I often come home mentally and physically drained. This impacts my personal life, leaving me with little energy for meaningful activities and instead defaulting to habits like watching mindless TV or simply feeling “not in the mood.” Many of my colleagues share similar struggles.  Over time, I’ve worked hard to stay current, learning to interpret and apply new technologies in clinical practice, which has expanded my scope of responsibilities. Despite this added expertise, I am not appropriately compensated for these advancements, unlike other professions. In fact, my income has decreased, partly due to the oversupply of optometrists, leaving me in a position where I am effectively doing more for less.  I wish our profession had a union or protective body—similar to the medical board—that could advocate for fairer working conditions and proper recognition of our value. | | |
| 3 | | 1. We have weekly meetings and are able to bring up issues which is really good thing. However, often my manager will find my suggestions at meetings threatening, because they didn't generate the idea themselves. This is a probably a personality/power issue and usually leads to a tense not discussion.  2. As the only optometrist in the practice I cannot take long annual leave breaks as we no longer have cover for my time off. I have to take shorter breaks therefore as Locum cover is too expensive. We are looking for another optometrist but this may take months or years as we are a regional town.  Suggestion: Your study could have asked if we are working city/regional/remote/ so that you can separate big city and regional responses. I imagine there are very different pressures on optoms in different locations. | | |
| 4 | | 12 months ago I was working at Specsavers and my answers would have been completely different. Specsavers is the absolute devil!!!! | | |
| 5 | | After a not so great end to my first job out as a optometrist, I changed to another corporate store (SS) with great directors and staff and feel my opinion and professional stance on things is valued. | | |
| 6 | | Alot of the negative workplace environment are the 2 big corporate practices in specsavers/opsm, and even among specsavers, it is only select stores. There is not much that can be done in giving more worker rights as the whole idea of capitalism is you are never paid what you are worth, and corporates have rigged the game in their favour. A piece of advice i got was, u either join the bullies or get bullied. The mate who said this now owns multiple corporate practices and does well for themselves.People expect to make a comfortable living without having to sweat capital or sacrifices on it, unfortunately, not many careers have that. | | |
| 7 | | Although most of the practice-centric responses are positive, these reflect my relationship with my practice team, rather than the wider company. Beyond the practice, I am greatly dissatisfied with my corporate-level colleagues, corporate values, and their processes (EssilorLuxottica). | | |
| 8 | | Answers would be significantly different (far worse satisfaction) when I was previously at OPSM. | | |
| 9 | | as an optometrist of 35 + years , and valued experience, I feel very short changed that I am paid the same or less of someone with 5 years experience. I am merely working in the industry now as it is too late to change careers | | |
| 10 | | Best care is often compromised in order to prioritise business efficiency. There is a lot of stress to test patients faster and faster. On top of that, salaries have become insufficient to meet independent modest living. The workload and risk involved are far greater than other careers which pay much more. | | |
| 11 | | By opening more university places and flooding the market the attitude towards optometrists has changed a lot. We take all the legal responsibility while been told what we do can be done by anyone. Specsavers and opsm have been trying to devalue us for years. The remote optometry attempt shows this. I just want to help patients and I shouldn't have up sacrifice my health and mental wellbeing to make even more money for companies that don't care | | |
| 12 | | Corporate optometry seems to have a high rate of burnout, have been doing this for 7 years now (started in NZ) and probably at least a third of the optoms that I have known or graduated with have quit and transitioned to other industries. Have spoken to other colleagues from different universities who graduated at a similar time and one had only half of his cohort left doing optometry. For me I’m finding it overwhelming to be 20min eye tests but still being stuck doing pretesting or dispensing in between. Retail side especially seem to be really overstepping and I have basically been told to prioritise dispensing for patients (as in they expect me to do the dispense when there are dispensing staff available)over doing referrals/admin work. I think the industry needs to change as it is not sustainable the way it is now. | | |
| 13 | | Corporate optometry where I have worked since graduating has become increasingly retail focussed at the expense of patient care. Managers will base pay rises on unreasonable KPIs which involve the sale of glasses and billing of medicare items to increase revenue under the guise of patient care. This encourages over prescribing of glasses and over utilizing of medicare billing unnecessarily. | | |
| 14 | | Disappointing that the state of the profession has devalued.  There is no advocacy and protection to our profession.  You have the profession being squeezed from either end. Ophthalmology from the top and dispensers from bottom. AI will have soon ways to perform refraction and assistive diagnostic ability at looking at optic nerve head/ and many more areas . Once dispensers gain access to this refraction it’s all over. Optometrist will be employed by dispensers and treated with low pay and quality. Optometrist will be used as an assistant to an ophthalmologist for repeat scripts of medication. A 5 year course does not leave optoms much of a long term career unless they open up their own and compete against corporations that have decimated the market.  The pay for a consultation is so low considering the effort you put in.  Too many graduates and no balance. | | |
| 15 | | Don't want to skew your data but just work 1 day a week clinically (0.8FTE admin/other) | | |
| 16 | | Due to the nature of corporate optometry (bulk-billed appointments with spectacle purchases as the primary source of business income) and the capitalist mindset (unlimited economic growth despite a limited population, limited resources, and cost of living crisis), the pressures placed on optometrists to sell undermines their training as allied health professionals. It promotes quantity of eye examinations over quality, and creates a culture where it is disadvantageous to see more complicated cases as they may require more time than allocated in the diary and may be less likely to purchase spectacles (what the company actually cares about, which becomes more obvious when considering most optometry KPIs). As eye examinations are so closely tied to the purchase of spectacles or contact lenses, clients tend to attend multiple practices and feel entitled to an eye examination (even if they have had one recently, and present with no concerns regarding their vision and/or ocular health) - this limits the capacity for more urgent appointments, especially if additional testing (requiring additional time) is needed. Only particular optometrists can thrive - those who like the fast-paced nature of such a set-up (including "squeeze-ins", and no allocated time for administrative work), and those who do not check ocular health. In practices that typically have longer appointments and/or see more complicated clients, a gap is charged ; this limits access to health care for individuals who are socioeconomically disadvantaged. | | |
| 17 | | Employers including Specsavers, OPSM, bupa, etc, control the way optometrists practice through immoral KPIs, and they actively work to lower our salaries and rates. The managers and directors are bullies and treat us horribly. A lot of optometrists struggle with mental health as a result and leave the profession. But no one cares because they are also driving new graduates. It’s not normal to replace optometrists every 2 yrs. We want better work environment, control of how we practice, and most importantly, generous pay for our skills, responsibilities and university Masters degrees. | | |
| 18 | | Every minute of our time needs to be accounted for. There is so much emphasis on sales and KPIs and money we make per hour that sometimes I feel like nothing more than a salesperson not a health professional. This has worsened considerably over the past 10 years and will not get better now there is a complete overhaul supply and under employment of optometrists. I haven’t had a pay increase in over 10 years, not even CPI | | |
| 19 | | Feedback given to my directors in store regarding admin time or diary issues is not taken on board and we are told that’s how it is or “that’s how we roll.” My giving feedback was retaliated with the directors bringing up one time I went home early instead of listening to or understanding the feedback, and the optometry team no longer bother trying to give feedback because of the responses from the directors. They want to give the illusion of approachability but basically scare us off giving feedback in the way they take the feedback. My hours over December have been changed without consulting me first, and when I try to speak up I am met with legal jargon or it is implied I’m being inflexible when I can’t adjust my hours due to plans outside of work. I feel I have no autonomy over my workload and I often feel trapped | | |
| 20 | | Font style of this survey could have been a better one with good contrast. | | |
| 21 | | For 8 years our practice was a franchise, run by a local, respected optometrist. He retired and the store came under corporate rule, so since then most of the experiences, qualified dispensing staff have left and I'm the only permanent optometrist. They do not put new staff through the dispensing cert programme like our previous owner used to and the provided training by head office is minimal. For a while there was direction from regional management to turn away red eyes or similar appointments which I explained is against an optometrist's duty of care if we have time to see them. I was appalled that this was even given as a direction. This unethical pressure/direction from head office lead to the resignation of our store manager of 14 years and I almost left, but thankfully the regional manager changed and we could see patients as needed in an appropriate time frame. They then put me on a PIP to improve my handover to the dispensers which was a huge waste of time and resources considering every other PIP within the company has been for patient safety concerns, malpractice and breach of contract. The practice is understaffed in comparison to when we were a franchise and the store manager runs 2 stores and helps at 2 others as so many staff have left in the region in the last few years. Hopefully conditions will improve on the long run. | | |
| 22 | | Full time locum currently. Was a full time employed optometrist previously. Responses are based on experience from both roles | | |
| 23 | | I am a practice owner and have had the opportunity to do a lot in my short time as an optometrist. I support my colleagues in their struggle to find their work life balance, as I have faced similar challenges earlier on in my career. However, having overcome all of that, I feel I may be an outlier for this survey. With that said, I did decide to complete the survey as I do hope my answers may benefit the greater optometry profession. | | |
| 24 | | I am considering a career change due to finding optometry stressful. I have almost an ideal workplace so this is not the reason for the stress, it is more internal. My biggest complaint about our qualification is that it is difficult to find work of a different type. | | |
| 25 | | I am fortunate to work in a great practice with very supportive, experienced colleagues and staff. Unfortunately due to poor pay rates for dispensers we have high turn over as people leave for better paid opportunities. The support and communication from higher up in the business is poor and lacks understanding and support for the challenges faced by our staff on a daily basis. Targets are increasingly unrealistic and optometry KPI's can be very demoralising for many of my colleagues who work very hard but don't measure up to corporate Optometry KPI's. | | |
| 26 | | I am fortunate to work in a practice that prioritises the clinical well being of our patients over commercial priorities. In addition I work with a dedicated and professional team of people. | | |
| 27 | | I am seeing a clear trend as everyone else that wages and working conditions of optometrists are declining. With this the wages are low than professional care and service level optometrists provide; but not just because of the oversupply in some regions i.e metro cities and more control by corporates but also the quality of work and "standard of care" acceptable in many practices (independant or corporate).   While a harsh sentiment, I feel not all optometrists deserve a very high paying job if they dont actively practice full scope optometry. Overall, the standard of care needs to be improved across the board as well.  To address working conditions, an overall minimum standards such as "Professional Award" or union-led EBA is needed whereby there is reward for work i.e overtime, weekend pay, late night pay as well as right to leave and holidays during busy periods. | | |
| 28 | | I am so upset with where the profession is heading Salary has been stagnant  Eye appointments have reduced from 30 minutes to 15 minutes  There has been alot of fraudulent health fund claims in all businesses  There has been plenty of mis or under diagnosis when I assess patients. This is due to the reduced time to provide a comprehensive service to patients. | | |
| 29 | | I am very happy in & always have been happy working in independent practice. A store I once worked for sold to G&M & it was awful. | | |
| 30 | | I am working at my third job since starting in the work force in 2022. | | |
| 31 | | I changed to an independent, well established optometrist about 6 months ago and am very happy. Although the practice is much busier, I feel heard and there is minimal politics. I have worked at several corporate owned practices where I was made to feel unimportant and ignored when issues were brought up or constructive criticisms were given. | | |
| 32 | | I dislike the policy of only being able to use annual leave in blocks of 1 week, it leads to wasting leave when I only need a single day off. | | |
| 33 | | I do not practice in corporate optometry by choice. | | |
| 34 | | I don’t think this profession is headed in a good direction at all - less and less about eye care and more about sales. Optometrists are pressured to give rushed or lower quality care because the higher ups are pressuring them to squeeze in as many appointments and convert as much as possible. The job market and security is abysmal. Absolute oversaturation of optometrists. Most people don’t want to or are unable to uproot their whole life and move to a rural location or interstate for a job. Most Optometrists I know of or meet are deeply unhappy. | | |
| 35 | | I feel as a profession we are undervalued and underpaid for the study and learning required, the day to responsibility of people’s sight and general health and our role in the community. I feel there is a lack of support from the profession to better our remuneration and Medicare rebates are the starting point for that | | |
| 36 | | I feel like nowadays，most of the stores are mainly focused on making sales，and KPI. which drives optometrist to be distressed of getting good conversion rate，so they have to minimize the number of pathologies they see each day. | | |
| 37 | | I feel that after 5 years of study, this career just hasn't been worth it. I feel very overworked, it is clear that us optometrists are not very well looked after, yet somehow we keep making sacrifices to show up on Sundays, Friday nights etc. I would prefer to apply my skills from my degree, have better scope of practice, rather than be pushed to "convert". | | |
| 38 | | I filled this out for my current role as a locum working in my family business, so my current job in optometry is actually quite comfortable, however it does not reflect the role that I had upon graduation, and I acknowledge my experience is a very rare one and unlikely to be the norm amongst respondents. If I were to fill this form 4 years ago as a new graduate, it would be very negative in terms of my experience in autonomy, workload etc | | |
| 39 | | I got lucky with my job, I've always preferred regional work due to salary and variety of patients seen. I do worry that I'll never be able to work in a metro setting due to the oversupply of graduates. | | |
| 40 | | i hate working for specsavers | | |
| 41 | | I have a lot of job satisfaction and my colleagues are also my friends. I work in private practice, and in a hospital. No amount of money could convince me to work in corporate optometry; I value professionsl integrity and my own well-being. My friends in corporate are burned out and could be replaced in an instant. Some of my private patients are third generation at the practice; I know their families and friends. Our advertising budget is exactly zero - we are fully booked. I am very lucky, I know optometry is under a lot of pressure. | | |
| 42 | | I have a single 20 minute paid break in my 9 hour day which includes my tea break but is referred to as my “admin” for administrative tasks  While my practice could accommodative new patients this is more due to increased clinical hours, rather than demand for eye tests having reduced | | |
| 43 | | I have a supportive store manager- but he has very little power or control of our systems. Working in a large corporate practice- all systems, policies, rules are made from head office. They often don't reflect local differences in our practice style, patient base or needs. Eg We have an elderly patient base who like to have appointments early in the week. But head office won't staff the practice appropriately because they think the main demand is always on a Saturday- not for us. Despite multiple requests for appropriate equipment such as an OCT, given our older patient base- always refused. Very frustrating. | | |
| 44 | | I have been full time in this role for several months, however the first 2 years were part time. During that time, I was also a locum 3 days a week. My answers would be vastly different for the majority of workplaces I worked at as a locum, particularly within Specsavers stores. I will be submitting a secondary survey based on that experience and will refer to this in the comments of that survey. Thank you! | | |
| 45 | | I have decided to leave my current role and take a part time position next year because of poor communication and culture problems at my current job. There have been more than 10 practice staff leave in the last 2.5 years I have been here. I believe that management and culture issues in optometry exist in both independent and corporate settings. The lack of job opportunities in the sector at the moment also make it difficult to negotiate better workplace conditions. | | |
| 46 | | I have worked for many employers in my nearly 30 years of practice. Conditions vary dramatically even between practices of the same corporation. Several employers I would never work for again, and the same goes for pretty much every corporate practice which I believe are ruining my profession with too much influence on the industry. | | |
| 47 | | I locum and I also co-own my own practice so my feedback is not necessarily the norm. | | |
| 48 | | I may be echoing the frustration of my colleagues and myself in this matter - but I was once extremely happy at my practice, yet this has all changed very recently.   We were an independent practice that was sold to a larger independent franchise. We were told nothing would change, however we already started to see small changes come through. This became extremely apparent when we were sold off at the beginning of the year to a large international corporate. Since then, we have had zero autonomy in lens choice and our special interests are not encouraged. We were a practice that other practitioners would refer to as we were well adept at behavioural, neuro and myopia control. Since then, we have had to turn away these patients. The staff are all leaving or have left and those that remain show signs of burnout. One of the main reason that staff resigned was due to burnout, both physically and mentally. This is sadly a very real trend in optometry at the moment. I know of many colleagues who plan to leave the profession, or have taken months off to deal with health problems due to burnout.   Some of my patients I have seen grow up have expressed wanting to be an optometrist. I have not deterred them from the profession, however I often think if my daughter told me she wanted to be an optometrist, I would gently steer her away from our profession. | | |
| 49 | | I often feel regret over choosing to study optometry. I am enjoying the work less and less. I really hope that we can make a positive change to this industry. | | |
| 50 | | I operate in a practice where patient care takes priority. Yes I am often very busy but there is always ample time to bring patients back for follow-up when required. | | |
| 51 | | I really enjoy working at the ophthalmology practice I work for and I think if the surveys were split into two complete different surveys - one from my corporate practice vs ophthalmology practice, my answers would be on the opposite ends of the spectrum, but it's evened out in this survey overall. I think the biggest factor in optometrists unhappy in this profession is that there's a salary ceiling with no further career progression pathways. Although I can't imagine the coprorates giving higher pay even if our scope of practice increased (e.g. therapeutically qualified optometrists get paid the same as ones that aren't). The number of new graduates that are added to the workforce also doesn't help with this capped pay and a lot of optometrists are scared to speak out due to fear that we are so easily replaceable. I think as optometrists in coprorate practice we are often pushed to see every patient without any breaks/ admin slots and creates a lot of burn out to the point at a practice I left I was told I ran late and if I take another lunch break that would cost the business $330 in sales. I think this sort of mentality degrades the profession and what we do in the core of it all as primary eye health care providers. | | |
| 52 | | I think optometry needs to broaden our scope into the hospital setting where we support and triage patients and provide more efficient ophthalmology type hospital services. This allows optometrists to practise more in line with how they are trained clinically rather than be just a refractionist. Hospitals may potentially offer better pay too due to reducing the need to pay for consulting Ophthals for tasks that optometrist can do . | | |
| 53 | | I took 2 months of "stress leave" which then became 4 months. I went back and nothing had changed. Working conditions are unreasonable. I have developed lichen sclerosis and dry eye making work even more challenging. | | |
| 54 | | I work as a contractor for an Ophthalmologist in a regional area ands have done for 11 years. The scope of practice means that I am managing either solely or collaboratively a broad range of pathology every work day in every patient I see. It is a fantastic role. I work one to two days a week in retail optometry typically for a “chain” to supplement my income. The retail optometry work is far less satisfying | | |
| 55 | | I work as a managing optometrist and therefore have more sway of decision making than otherwise, so my responses will be skewed. | | |
| 56 | | I work in a mix of independent permanent part time employed and locum at corporate (Specsavers). Independent PPT: financially feeling the squeeze and loss of patients to Specsavers. Workload is fine, but I'm on the same salary I was commanding 10 years ago. Salary stagnation due to a dramatic oversupply optometrists is stark. Locum: work is now scarce but I'm fortunate to have rapport with enough stores to keep me in (just) enough work | | |
| 57 | | I work in a well established private practice with excellent support around me. I do feel anxious and nervous asking for a higher salary despite taking on managerial tasks like rostering of staff. I do see through various media, a growing dissatisfaction within the optometry profession about career outcomes. Sadly this discontent has continued to grow since I graduated in the same year as a newly established “accelerated” optometry course. Since then two, and now possibly a third course, have opened and the amount of graduates does not seem justified. As someone in a secure position I am still concerned about the churning through of new graduates and what it means for our profession. | | |
| 58 | | I work in the pharmaceutical industry due to poor work pay in mainstream optometry. I locum in optometry to keep up my skills but am glad I’ve made the transition as optometry appears to be going downhill with our time and skill not appropriately covered financially, and increasing KPI pressures from corporates that value spectacle sale KPIs over patient care. I would like to see this change with optometry moving towards a dentistry like model with appropriate fees as Medicare will never pay what we are worth. We need to catch up with oral prescribing and increased abilities such as AMD injections, however we need to ensure this is compensated for when increasing our skillset. Our ocular therapeutics is not valued by Medicare rebates or employers and this needs to change.   We also need more protection and support for optometrists and working rights with the corporate takeover and the way optometrists are being treated.   Many of us are starting side gigs/other jobs/changing professions as it is too difficult to financially afford a family and the cost of living now with decreased optometric wages | | |
| 59 | | If I am fully booked, or even close to fully booked, let's say 12 or more appointments a day, I have no time for admin tasks, I also find it hard to 'help at the front' as requested by the company. There is a misconception that if I am 'not with someone' that I have nothing to do. I find that I have to do everything in the consult time to avoid those conversations later on that there is some admin to do.  I have worked in a practice that allows 20 minutes and no pre testing (most of the time pre testing is not that beneficial) and I am ashamed to admit that I have cut corners and done a sloppy job. These practices typically book in 20-25 appointments a day. I feel I am not rewarded for the amount of turnover I generate for the practice. Not to mention the physical impacts: sore shoulder and back, no time to go to the toilet or get a water.   Otherwise Optometry is very satisfying. I think 8-12 appointments a day is the ideal amount. I can do a good job and have time for admin and a short water break. | | |
| 60 | | In my particular circumstance, I feel I have quite a good work environment regarding the people I work with, and my voice being heard. What I do find I struggle with is the mental tax that occurs when seeing a high volume of patients, especially as we only are allocated a 30 minute lunch break, and no other 10 minute breaks during the day as other retail staff members get as part of their award. The mental tax adds up throughout the week and I am usually exhausted at the end of the week. | | |
| 61 | | In my work as a sub contractor at a corporate practice it is overall a negative experience, on the contrary my part time work in an ophthalmology practice is an extremely positive one with lots of support, open discussions and opportunity to grow | | |
| 62 | | in the process of leaving to go to an independent practice, for improved QOL at work. | | |
| 63 | | Increased responsibility with introduction of therapeutics has not been matched with increases in remuneration. We have much more responsibility but are not compensated accordingly. | | |
| 64 | | Independent practice in a regional town can be a trap if your employers are difficult to work for and there is a high staff turnover. I just hope in this regional setting the high staff turnover doesn’t reflect on me as an optometrIst. | | |
| 65 | | Industry oversatured with graduates, which results in lower pay. Seems like larger corporates are the norm with finding a stable position as there are fewer independents and those jobs are in high demand | | |
| 66 | | Interestingly, I found that many of my front-desk/dispensing colleagues have somewhat negative job satisfaction due to a number of issues in current higher management and supplying operation. | | |
| 67 | | It is disheartening to constantly be faced with KPI pressures and to year after year have your performance based on sales. However it seems three years into my career, I have already hit the ceiling in terms of career development and salary level. Where to from here? It is tricky to branch out when there is little movement in the market/ any opportunities often come with a significant pay cut. I feel our peak bodies need to be doing more campaigning for possibly standardised pay levels/ pay guides based on experience. The universities also need to stop letting in so many students, we are so oversaturated in metro areas, and I have some peers, less than five years out, who have been made redundant, only to be somehow ‘replaced’ with a graduate who is much cheaper. Most of my friends at the three year mark have been encouraged by employers to ‘drop to part time, as the practise can’t afford them full time’. Soon rural positions won’t be in as high demand either and then where will all the optometrists who are on 100k or more go when our employers can hire graduates for cheaper pay? I feel let down by the universities, the corporations that dominate the market and by our peak bodies. | | |
|  | |  |  |  |
|  | | |  |  |
| 68 | | It is tragic how the industry has fallen so much over such a short period of time. The reality is that compared to the older generation optometrists who lived through the 'golden age of healthcare', the working conditions for ‘the average optometrist’ have progressively been on the decline to the horrible state that it is now and optometrists these days don’t even get compensated with the job security or renumeration that is anything close to what it was like ‘back in the days’. There’s probably a lot of young optometrists who chose the profession as they saw what it was like for the older generations and currently feel betrayed as the profession has significantly declined in value by the time they finished their studies and started their careers – it is ironic how they are the ‘more qualified optometrists’ but are also the ‘less valued and less compensated optometrists’. All of this while having to meet employers’ crazy demands of being an optometrist who can also be a dispenser, working under extreme conditions where patients are booked back-to-back with no breaks and optometrists are often expected to squeeze in extra convertible patients or emergency patients on top, fighting to have a proper lunch break or go to the bathroom in the midst of all the chaos, all of this while trying to pay off HECS debts with such little salary, no wonder why a lot of optometrists are burnt out only one or two years into their career and end up wanting to leave the profession that took 5 years to join. And the ones who are staying are not doing so because they like the profession, but because they are already 10-15 years into their career, they have kids and a mortgage, and it is too late for them to pursue anything else.  While I think that it is really the giant corporates that played the main roles in the demise of Optometry as a profession, I can't agree with people who believe that having more independent practices or replacing corporate practices with independent ones is necessarily the answer. Yes, I have heard of plenty of optoms who had horrible experiences and left corporate practices, but I have also heard of just as many optoms who had the same experiences with independent practices, with some of these people resorting to extreme working conditions in their new roles because that's still better than having to work at their previous workplaces. While I know of great optometrists owning/working at independent practices, there are also some that I found aren't providing the best care through old patients of theirs that came to me. I heard last year that a graduate role at an independent practice all the way at Sunshine coast was advertised to Optometry students with only an 80K salary. I have also come across a LinkedIn post by an optometrist who was claiming that “corporate optometry has created an oversupply of optometrists in order to reduce salaries for employed optometrists”, which I agreed with, but then continued to push this message that this is why everyone needs to own an independent practice so that they can take advantage of cheap graduate rates. It seems like there is at least a proportion of independents, whom just like how people often says for corporates, don't appreciate optometrists, don't treat optometrists fairly, don’t pay optometrists any better, and don't necessarily provide better care for patients than corporate practices. What I want to say is, while corporate companies as a whole have had significantly negative impacts on the optometry industry, on a practice level, not every independent practice is good and not every corporate practice is bad to work for and I would like to see universal regulations put into place so that no matter what type of practice an optometrist works in, they will be treated appropriately with a fair wage that reflects the skills, expertise and profit they have studied and worked hard to bring to the table.  Something that I think is worth pursuing to improve working conditions for optometrists is to introduce new job opportunities. As of now the places available for optometrists to work in are mostly corporate and independent practices and ophthalmology practices, and there is currently an oversaturation of optometrists where graduates have to compete with each other on the few metro positions available every year. This leads to optometrist taking whatever lowest salary they could to make themselves appealing to potential employers so that they could remain with their friends and family, and not have to live in a location where people can feel vulnerable or unwelcomed to live in, considering that majority of Optometry graduates these days are not Caucasian and are female. In summary, optometrists are currently competing for work positions rather than employers competing to recruit optometrists to work for them, which has put optometrists at a vulnerable position. I would like to see more job positions, not just quantity but also in diversity, so that existing employers will have new contenders to compete against, which should lead to better compensation and better treatment of optometrists. There are several examples where this has been successful for other health professionals. For example, pharmacists now have the option to work in hospitals and in research settings (e.g. Clinical trials) and I have been told that this led to existing employers offering better compensation to pharmacists and that pharmacists were overall more satisfied with their profession. I think by having more diversity in job opportunities, it creates competition between employers to recruit and retain health professionals and also gives health professionals an alternative option to consider if they are unhappy with their current workplaces. At the moment, a lot of optometrists are having to put up with a lot of things because they feel like there is no other choice – the only choice they have is between staying at their miserable jobs or being unemployed.  Personally, I haven't had the greatest dissatisfaction with my own workplace – sure it isn't the most perfect place to work with and it does come with its own flaws, yet I still found it to be better compared to a lot of other places that I have worked for in the past and compared to other places that my colleagues have told me about. However, I am currently leaning towards leaving the profession because of my dealings with Optometry Australia. I have been involved in a certain OA group (I will not say exactly what as I wish to stay anonymous), but I found it to be a truly disappointing experience that I would like to briefly share about. The purpose of the OA group was to support other optometrists and there were other members of the group both within and outside of OA employment who supposedly joined the group with the same intentions. However, I came to realise quite quickly that the group and its members didn’t actually have optometrists’ best interests in mind. I felt like OA’s main goal for this group was to use it to retain existing members and gain new members, and also gain more interest for their events, and while I believe this can certain be a side goal or an interest for OA to have, it shouldn’t have been prioritized over the original purpose of the group. As for the other members of the group, I came to realise that a lot of them are just using this group as well as the Phoropterfreefridays Facebook page as a platform for them to promote themselves to gain popularity and make a name for themselves in the industry. I can't go into too much details but there are some individuals within the OA group that are also on the Facebook page and these people will talk on the Facebook page as if they want to stand up for other optometrists and they're trying to advocate for optometry to be better for everyone, while in the OA meetings they will be blatantly condescending of everyone in the group and all of this was just disappointing to see. When I tried to bring attention to the real issues that optometrists were facing, all I got was a super long silence, some people raising eyebrows, and one of the facilitators of the group saying ‘thank you for that, we're going to end the meeting now’, and this topic was never brought up again in future meetings. I felt like I just got treated like a crazy person when I was trying to make a difference, and I felt like this battle is not possible to fight on my own and thus not worth continuing. I’ve learned to just keep my mouth shut in group meetings and I am currently just waiting for my term in the group to end and to move on from the profession. It's a bit ironic that me joining a group that was supposed to help other optometrists was what led me to want to end my career as an optometrist. | | |
| 69 | | It’s disappointing to see that optometry has become very corporate and that my value as an optometrist is determined via KPI‘s which are fundamentally related to moneymaking as opposed to patient care. I find it frustrating that I have to answer to a regional manager with very loose morals who expects me to compromise my integrity just because he finds it so easy to do so. I find it disheartening that optometry salaries simply do not keep up with inflation. It has become a very unhappy and undervalued profession. Let’s be honest, it’s very boring too. | | |
| 70 | | It’s hard to be passionate about your job when you feel like you’re just there to make corporate owners richer, and aren’t supported to provide best care. This leads to burnout being experienced more easily. | | |
| 71 | | I've just recently moved from corporate to independent, best decision I could have made. | | |
| 72 | | Job opportunities/vacancies for optometrist work seem to be less compared to previous years. For example, when looking for work, there are less choices for the type of practice, location of practice, locuming work etc.   It can be quite challenging and tough for Independent practice/smaller business as the industry becomes competitive especially competing with bigger corporate companies such as Specsavers, such as struggling with not enough number of patients, price of products etc. | | |
| 73 | | Lack of career progression opportunities.  Low salary ceiling. | | |
| 74 | | Lack of job security is a concern Glad that I have enjoyed the earlier days of optometry 20 years ago—but it looks bleak when comparing back. Way too many new graduates and now 20min appointments have become standard as well as waiting a week for new spectacles. Low vision is barely an optometry concern. | | |
| 75 | | Large and icreasing number of optometry schools, corporate practices, and influx of graduates make the profession less valuable. Most optometrists reach a salary ceiling very soon which in not proportionate to thier investment of time, money and expertise. | | |
| 76 | | Learned the hard way the importance of having a boss who is an optometrist and therefore understands the finer issues of managing patient care and outcomes, the need for an optometrist to have time to prepare letters and reports, and that best practice is not always selling a pair of glasses to someone whose eye health is not going to result in better vision with a minor change in rx. | | |
| 77 | | Locuming is the best of you don't mind the lack of job security | | |
| 78 | | Looking at the current situation, optometry is not worth pursuing anymore. I wouldn't recommend anyone to opt for this course. | | |
| 79 | | Majority of my dissatisfaction with optometry are linked to excessive KPI pressures, particularly encouraging same day spectacle purchases. There is a heavy emphasis on conversion and as a heavy influence to push small Rx changes as something essential for patients and that they must changes their glasses. Low conversion performance is often accompanied by sit ins to ensure 'the right language is being used inside the test room' and not let opportunities slip by. I feel my ability to give an honest answer to patients asking if they should change their spectacles following small rx changes is significantly impacted in a negative manner. It results in being reluctant to see patients who only present for routine check ups with no symptoms and reluctant to see patients who are considered a 'red appointment'. In my early career it was a significant source of stress and caused a strong drop in job satisfaction where it seemed value was not placed on my clinical knowledge and capability, but instead on KPIs. Over time I have shifted my mentality to undo the effect of this KPI pressure on me, and I strive to see and treat all appointments as the same and now simply ignore the KPI pressures on me. I would be lying if I said this is easy or that the KPI pressure still isn't felt, though I do my best to not let it affect my practice. My issue remains with sit ins when they happen. It's not common, but can still happen. If it were only a matter of seeing appointments and doing what I feel is best practice with no KPI pressure, my job satisfaction would sky-rocket. For my own mental health and to avoid poor job satisfaction from affecting patient outcomes, I do my best to avoid bending to KPI pressures and not worry about it. | | |
| 80 | | Mandated tea and afternoon breaks and time for referrals would be appreciated on days where there is a full appointment book. | | |
| 81 | | Many of these questions are quite ambiguous. In the current state of the industry it feels as though we are unappreciative of what we earn, but are made to work harder with little say. All under the unmentioned “threat” of replacement and lack of other jobs. | | |
| 82 | | More emphasis from the association on increasing medicare rates in alignment with what we do. The association is absolutely PATHETIC in pushing the optometry barrow and actually achieving an outcome. Case in point, the lame introduction from the new presiident spouting the same old rhetoric we have heard from EVERY one of his predecessors. The assocaition is a disgrace and has let the corporate entities dictate the number of graduates to the ponit of massive oversupply. The Association is selling young, bright students a dummy with Optometry as a profession - do 5 years study, get a massive HECS debt and then, if you are lucky, get a job with SpexSavers/OPSM for minimum wage and crap hours with the prospect of being replaced by AI in the near future. Good job there at Head office, congratulations. | | |
| 83 | | My bosses are not good at retaining optometrists. The last 5 optometrists that joined, all left as they were declined pay rises. | | |
| 84 | | My current work place is in regional Queensland and I work both weekends, hence the slightly higher than average? pay with regional and weekend loadings as a 2nd year optometrist. However, from this year, as a 3rd year optometrist, I will be relocating back to metro and will experience a reduction in salary due to absence of regional and weekend loading, so just FYI. | | |
| 85 | | My Locum jobs are high pressure- to sell spectacles and no time to do reports/referrals. The equipment is old- no OCT- VFT is ancient | | |
| 86 | | My own store manager involves me in decisions and values my input. However most major decisions are made by head office and head office does not ask nor care for my opinion. | | |
| 87 | | My practice is better than some in the chain I’m employed at. The individual managers are more important that the company policies. | | |
| 88 | | My practice team is fabulous.  Practice manager works between locations and isn’t really helpful to me  Regional Manager is not easy to work with, doesn’t take feedback, doesn’t accomodate leave in black out period (mid Nov to mid Jan)  HR is not approachable and won’t take feedback into consideration (just ‘that’s policy’) No pay rise in over 5 years as not meeting target as a practice.  Some days are very quiet and can easily take more patients and emergencies. But can and will be overbooked if full book.  My reviews can be 10 or 20 mins. I put 20 in the survey | | |
| 89 | | My practice was purchased by a large corporate group. I now have a non compete hanging over my head or I have to move town. I live regionally and moving my young family is not an option.  I am doing more work than ever but am now getting paid less than I use to due to changes they have implemented which are in breach of my contract.  I have talked to our advocacy body OA which have advised is not worth challenging because they are bigger.  I feel powerless in my role and can’t make a career change at this point in time. | | |
| 90 | | My previously independent work place was taken over by corporate. The feel has completely changed and it has lost its unique flavour. I am looking at starting my own practice in the next 12 months. I am sad to see the changes in the independent optometry world. | | |
| 91 | | My relationship with my optical dispenser qualified boss is what makes me enjoy my work so much considering I work for corporate | | |
| 92 | | My workload is manageable where i am at this time. What is not manageable are the KPIs. I am vonstantly just based off of ridiculous metrics such as conversion and AOV when these have very little relevance to patient care. I am being constantly told that I must recommend patients change their glasses over small Rx changes and that these are the best care for the patient. This makes feel like I am outright lying to patients when I know that if I rechecked them hours later or the next day, that they would have yet again another small Rx change. Its utterly ridicuous to me that I am being made to feel so much pressure to prescribe these small changes on to patients, feeling like Im a glorified salesman. I alreadt work in a low socioeconomic postcode and many of my patients are unable to afford new glasses even when there is a clear large Rx change. Many of them express worry about how they will afford it. I feel it is extremely tone deaf for optometrists to be pushed to prescribing these small Rx changes knowing full well it will make no difference to their outcomes, especially at a time when there is a cost of living crisis. We are being put through a series called 'trusted experts' which attempts to address patient objections to small Rx changes with ridicuous language modifiers to convince them that it is worth changing their spectacles. Following through eith this makes me feel like I am outright lying at worst and at best strongly misleading patients about their eyesight. I truly believe that majority of those pushed to buy new glasses over these small Rx changes would be furious if they knew the true scope of what was involved. My first priority is patient autonomy and I now flat out avoid these language modifiers I have been taught. Forbidden words to say during a consult are 'update if you want to, your choice'. Im supposed to 'be the expert' and tell them they must. Well, now I always ever say 'update if you want' to patients with small Rx changes, otherwise I cant sleep at night knowing that I at best misled them into spending potentially hundreds of dollars for very little gain. Its shoxking to me that the industry has become the way it had unchecked. If the public were aware of the true scope of such behaviour, optometry would have a severe crisis and loss of fiath by the public, rightly so. I fear for the future of the profession if this continues unchecked and seriously has me considering leaving the profession for good. Years of my life and thousands of dollars in hecs still unpaid for absolutely nothing. While private companies continue to rack in record profits, our salaries continue to stagnate. The future of optometry looks very bleak indeed to me. I have no faith whatsoever that things will change, by AHPRA or by OA. | | |
| 93 | | My workplace is good  Our profession is going down. There needs to be public awareness of optometry's lack of Medicare funding and normalcy to private billing advocated to the public With corporations downplaying this by oversupply of optometry unnecessary optom school intake numbers and schools numbers itself, lots of colleagues are unable to pay bills. For a degree where entry is bright scores and lots of brainpower to get through a long degree let alone financial burden of the degree only to come out for a career that their is no public respect and patients looking pay zero due to marketing of "free " exams by corporates and substandard eye test times to accommodate for this, whilst independents can no longer survive | | |
| 94 | | need higher pay and less graduates in the profession | | |
| 95 | | Need to increase salary of optometrists. I work in an independent practice in Melbourne, doing full scope optometry (paediatrics, specialty contact lenses and vision therapy). There is an emphasis on high quality care with building rapport with patients. I charge for scans and private fees (on average on $130 for initial consultation). Yet my salary $84825 (4 years experience) is significantly less than a corporate optometrist at the same level or even less than a second year graduate working at specsavers. My boss mentions that the salary ceiling in indepedent optometry is $90,000. This is unacceptable, as I am expected to practice full scope, take in complex referrals from other corporate optometrists and give high quality services to differentiate as an independent optometrist. There are other careers that don't need a university degree that pays better, and I'm probably better doing night shift at woolworths. Optometry Australia is like the Labor Party, too focussed on a leftist agenda and vouching for the elites (e.g. optometry practice owners, corporate CEOS, high credentialled optometrists). It does not support the rest of us "average joe" optometrists. Worse, is that I pay $2300 yearly for OA membership, $300 yearly for APHRA member and also the $4000-5000 for the optometry equipment. I have a huge HEC bill, yet my salary does not match this. | | |
| 96 | | Not enough power for optometrists compared to retail. Retail partners and managers don’t care or consult optometrists about walk ins or matters related to patients as to retail such as new symptoms. Unable to discuss issues which may help the flow of clinic or overall improvement in outcome of patient because it’ll affect the business financially.   Pressures with KPI and faster testing time are unrealistic. Practice seems to value sales over true ocular health care, despite the practice having accessible ocular health as part of their vision. Unable to develop strong and long term relationships with patients cause there’s no time at all to talk to them. Feel burnt out easily, tired and exhausted out of work, unable to muster energy to see friends/social events, or hobbies to keep me sane or alive.   Practices available for hire are too far away/regional. The pay for regional is not worth it; it keeps going down no matter the company. Pay is ridiculous in metro areas; 3-6 years of study for such poor pay is embarrassing. We as a profession are not treated as professionals, and our value and years of education are constantly undermined by staff we worked with, and patients. Moreover, our profession and income does not at all reflect the income we deserve as a profession. Patients understanding of what optometrist can do needs to be changed, including understanding of ocular health and co-management. Patients and staff need to have a changed perception that optometrists are not retail workers, rather a profession. There needs to be a clear disassociation between retail and optometry. Optometrists needs better Medicare benefits, which leads to them being self sustained without the need at all for retail sales. Scripts and prescription for glasses dealt with by the dispensing team. KPIs should not be sales focused AT ALL; rather, health and patient experience focused.   No available access to good equipment like topography to practice more broad scope optometry. | | |
| 97 | | Optometrist are over-worked, under-valued, under-paid and the scope of practice is not increasing at a fast enough pace to give optometrist the opportunities they need to have a fruitful profession. The opportunity for professional growth or practice ownership has never been lower. Respect for optometrist amongst the community is diminishing by the day. | | |
| 98 | | Optometrists are micromanaged at my practice with a few examples: - Any appointments longer than 30 mins (even with appointment notes justifying reason) are often questioned by "higher ups" that don't work physically in store (sometimes with calls to the store to question) - We've been on compulsory "customer journey trackers" for years which we need to fill out daily (without blocked off administrative time), listing every patient we've seen, whether they were pre-tested (supposed to be by retail team but often by ourselves), reason for visiting, whether tech was done (optos/OCT, charged privately), our glasses lens recommendations (must have 2-3 recommendations), dispenser 3-way-handover was done to & whether they converted or not - Between any no-shows & gaps, we're expected to do our administrative tasks which include: clinical notes, reports/referrals, medicare rejects, noting ophthalmology correspondences, ordering own patient contact lens trials, ordering cupboard stock contact lens trials, calling patients with contact lens trials waiting for follow up appointments, Leonardo (compulsory online modules), ordering topical drugs & misc supplies (like multi-purpose solutions, plungers, lubricant eye drop samples), call "third-hit-recalls" (weekly uploaded list of patients classified as bronze, silver, gold, platinum depending on how much they've spent on their glasses the year before, to book in an appointment), help with front retail staff (handovers, arriving appointments, adjustments), take own payments for additional scans (optos & OCT charged privately) AND write notes in any existing gaps in the diary to show which from this list we're doing at the time to (justifying our time)  Hard work is not rewarded, optometry is not respected, and the scope of tasks involved in my job is no longer just about seeing patients. Burn-out comes in seasons, but it got to severity where professional helped was sought after externally. Optometry does not feel like a sustainable career. | | |
| 99 | | Optometrists are not paid enough for the amount of responsibility we have. Our pay is also affected by an oversupply of optometrists which is a major problem in our industry. I feel sorry for all of the new graduates who spent so many years studying and accumulated HECS debt and now cannot find a job. | | |
| 100 | | Optometrists are not valued, and our patients health is not valued. Any positive health outcome for my patients is the result of my own and solely my own effort and dedication to my patients at the expense of my own mental burnout. This industry and the monopolies running it couldn’t care less about patients unless they are buying glasses every year. | | |
| 101 | | Optometrists are only treated as factory workers whose sole purpose is to sell glasses. And that’s happening across independent practice as well as corporate. It’s an absolutely horrendous profession to be in and I think it’s absolutely unethical that universities are telling young students this is a healthy profession to be in and training them up in these quantities. The education system has a lot to answer for and academics who promote these university placements should be ashamed of themselves. Working at Specsavers’s is a special type of absolute HELL. | | |
| 102 | | Optometrists are trapped in having to provide high quality care with too short appointment times - we carry responsibility to not miss potential issues . We have a repetitive job with no connection to the outcome of our prescribing - no relationships with patients and we are paid less than a teacher or a nurse . The profession is overworked and underpaid and it is similar to being a robot , but a human one . Specsavers is the worst. I am in a practice that is not very busy so my location allows me breathing space . When I worked at Specsavers I felt burnt out and very stressed after every day of work . It was a living nightmare for 10 years . There is nobody to speak to about thaws issues or you will not be employed . I would not recommend anybody study optometry as it currently is in this country . | | |
| 103 | | Optometrists are underpaid in respect to the proportion of revenue they generate/enable for the business. This is particularly apparent in high volume corporate style practices. | | |
| 104 | | Optometry as a profession is extremely unfulfilling and unethical. I began my career at specsavers where I was overworked and had no time to provide adequate care. Each day I would have back to back 20 minute appointments, on top of which I was expected to “squeeze in” visual field results and contact lens fits. I recall one New Year’s Eve between 4 optometrists we were fully booked with back to back 20 minute appointments but the store managed to “squeeze in” an entire days worth of appointments on top of that between us. No one had a lunch break. You don’t have time to connect with patients, you’re so busy you don’t even get to talk to your colleagues.    Since leaving this job my working conditions have vastly improved. I work in a quieter clinic which means as it is a new clinic but that means more pressure from upper management who are constantly harassing us about meeting KPIs and sales targets. They are becoming more and more controlling of our appointment books, have recently dropped to 20 minute appointments like specsavers and are now moving to a cheaper lens manufacturer. There is no room for growth unless you want to own a store or be a manager, it is so isolating and it is so difficult to find anything else you can do with the degree other than retail optometry.   With AI becoming more advanced as well as a projected oversupply of optometrists thanks to all the new courses and lack of caps on university cohorts, there is less and less job security and wages don’t increase unless you move jobs. I believe the future is bleak for optometry unless there is a big change and I am planning on leaving the profession as soon as I can. | | |
| 105 | | Optometry has been oversupplied and devalued very quickly, supposedly they could do nothing about it yet 20 yrs ago the association and the optometry schools kept numbers low/kept wages fair. Optometry became too focussed on increasing scope of practise without pushing medicare to fairly pay a benefit for this skill...would be better off leaving medicare yet the Association too worried about access to eye care. Dont think the dentists prioritise if patients cant afford treatment...GP s simply won't bulk bill if they don't think it is adequate. Expected to have more skills and responsibility for effectively less pay. No pay increase over 10 years..cost of living increased so I am worse off. Constant talk from the Association about liaison with government and GPs, out of touch with those employed by corporate. Dentists and medical profession will stand up to the government much more than optometry...eg Care Plan payments to Drs...nothing paid to optometry. The students I work with trying to get onto medical school...I advise them there is no future in optometry...I hope you get into medicine,at least it will be a career. Will be leaving the profession in the future 1 to 2 years | | |
| 106 | | Optometry is a poor career choice for high achieving high school students. Remuneration is poor and there is no avenue for career advancement. High school students should be given this information when considering their university options | | |
| 107 | | Optometry is not respected for the work we do. Patients view us just as glasses providers and employers like Specsavers take advantage of this. It is all about selling glasses and not about patient care. I did not study 7 years to sell glasses. Promotions are based on how glasses we sell. We are overworked. We are expected to perform comprehensive eye tests back to back sometimes even in 10 mins if the patient is late or its a squeeze in (does not align with medicare). We hardly get our lunch breaks (assinged 30 mins even though should be getting paid 10 min ones too). They are always under staffed. They want us to do extra visual fields to bill extra medicare even though not required- all to make money for the business. We dont get any time to write letters or do admin tasks- have to do in our own time. They take advantage of us as they say there is no other jobs and hence keep our salaries low. | | |
| 108 | | Optometry within a corporate structure favors the employer far too greatly. The 'scheme' that certain corporates have come up with is utterly disgusting and they are taking optometrists for a ride. There needs to be greater support from organisations like Optometry Australia to work for the optometrist interest. We need a union! | | |
| 109 | | Our Profession is in decline and has been for many years. Control of the profession needs to be in the hands of the profession. Currently it is in the hands of large overseas based employers of optometrists as well as the spectacle frame and lens industry. (Not to mention control by Private Health Funds) We need true professional autonomy and not to be puppets of industry and vertically integrated employers with vested interests in selling their products and using optometrists to facilitate those sales.  We may need legislative change to protect our profession from such pressures - this would be in the public interest. The Medical Profession would not be controlled by supporting industries in the same way. It would not be tolerated. Why is it tolerated in Optometry? | | |
| 110 | | Over recent years the number of staff rostered on has decreased. There was a time when there were always at least 2 staff for the whole day when an optometrist was in store. Now in the practices where there is only one consult room it is common to have only one staff member on with perhaps a part-timer coming in to cover the lunch.  On more than one occasion I have had staff members come in to my room crying as they are overwhelmed by being the only person on the floor trying to deal with everything. Frequently they don't get a lunch break or have to wait until late in the day before taking it. They tell me that on days when they are in the store alone on non-testing days that they have resorted to peeing in the sink or peeing in a water bottle as they are unable to take a toilet break. Quite horrendous really. | | |
| 111 | | Over saturated workforce with salary growth that is stagnant. Truly a dead ended profession with little choice on where to practice | | |
| 112 | | Over the last 6 years in my optometry profession, I have worked for various employers and managers and have always felt that the quality of care I provide to my patients is not reflected in my pay, bonuses or appreciation. I feel that the retail sector overrides and many corporate companies refuse and attempt to reject emergencies for the sake of KPI which conflicts with my duty of care and my passion in ocular health as a health professional. I don’t find satisfaction working for others with this mentality and find my closest suit in independent optometry. However, I experience the pay cut in independent optometry and not rewarded as a health clinician which is why Locuming for me feels more worth it but unstable with the current market. I would like a work life balance with flexible hours. Higher salaries and autonomy to spend adequate clinical testing time with the patients would be most desirable. I’ve noticed optometrists in America are recognised as eye doctors and have a wider scope of practice with invasive procedures making them valuable in the eyes of patients. Australia should work towards this through our optometry association allowing optometrists greater authority and training required to prescribe therapeutically and perform minor procedures. We see the ophthalmology sector in private and public sectors over burned and one per state entry level of medical doctors into ophthalmology program every year. Yet there’s an influx of optometry graduates every year with comprehensive ocular knowledge, why then are optometrists not relied on to ease the burden in ophthalmology by increasing our scope of practice to accommodate for more invasive procedures that would incur great revenue?   I’m an optometrist who wishes to be empowered as a health professional and want to do more clinically without the strict boundaries of optometry. | | |
| 113 | | Pay has gone down in real terms. | | |
| 114 | | Pay is bad, there is no respect for optometry and no pay profession. The future is bleak for employed optometrists | | |
| 115 | | Prior to having a practice, I have worked in multiple corporate settings. I found that having reduced appintment times of 20mins made me feel rushed. I felt I needed to do all adjunct tests I need to do in one appointment (eg. Gonioscopy, dry eye work up, visual fields) which made me fall behind in clinic. If i were to bring patients back for extra testing it would affect my KPIs and that would be frowned upon by managers. As I want to do the best for my patients I will squeeze it all into one session and do my best to catch up. But over time this caused me to burn out. If there were catch up times blocked off the book it would make it more manageable. Or admin time at the end of the day to write reports. | | |
| 116 | | Profession is in crisis | | |
| 117 | | Re the questions about walk ins and emergency patients- I can accommodate them about 50% of the time, just depends on the day if the book is full. I am not expected to squeeze in extra patients if fully booked, fortunately.   One thing to note with corporate brands is that there can be differences between how each store manager runs the practice, despite general guidelines from upper management. | | |
| 118 | | Regarding appointment booking and walk-in patient questions, we have 1-4 dummy slots per day (depending on how many optometrists are working) to accommodate emergency red eyes only. If there are no emergency red eyes, these slots are used as admin time. If there are walk-in red eyes, these slots are used and therefore there won't be admin time scheduled for that optometrist for that day. | | |
| 119 | | Request for larger screen denied despite small font and discomfort. Windows covered with advertising decals blocking view out making it even more like a box. | | |
| 120 | | Retail team often undervalue an Optometrist’s role to provide clinical care and we often feel pressured to overwork to pump out prescriptions. Workplaces pressure Optometrists to not call in sick and only schedule annual leave when it best suits the practice. | | |
| 121 | | Some of the questions were worded as if decisions on how to run the practice are made at a store level, whereas there is a big difference between stores and higher up levels in terms of expectations and work conditions. | | |
| 122 | | sorry - I was unable to answer a number of the questions, given my role is essentially non clinical | | |
| 123 | | Specsavers bulk bill all appointments. Private practice I also work in charges a lot on top of Medicare for OCT and biometric scans. | | |
| 124 | | Specsavers limits treatments I prefer work with many facets , managing dry eye , binocular vision treatments , knowledge of optics variable with dispensers , miss seeing trendy better quality frames , give too much away free, cl teaches, aftercare, | | |
| 125 | | Thank you and I really appreciate your team making this survey. I am very keen to hear the results. It is nice to finally feel seen and heard.   As a graduate I was made to feel that a corporate job was my best option and only option. Once I started working, I felt trapped in my current role due to the fact that if I left within 24 months I'd have to pay my bonuses back in full including the money that had gone to tax and super which I no longer had. As I also relocated as part of this role, they gave me some money to use for those costs, but the costs were far beyond this amount and started to eat into my bonuses. If I left I'd also have to pay back the relocation allowance, AHPRA fees and CPD allowance (which I used for my OA fees) to them, so overall I would effectively be in debt if I left. The financial stress / fear of this, combined with burning out at my job but not being able to leave, had a significant impact on my mental health. In a few months I will finally be free of this job but another grad will be quick to fill my place. Someone needs to warn the grads and protect them, we are all burning out before we even get our careers started. | | |
| 126 | | The job market is heavily oversaturated.  KPIs are causing high staff turnover and burnout. I am tired of every single appointment being micromanaged, and being told that it's only my workplace doing this, when it's on an industry wide level.  My work conditions are toxic, with no time off allowed without significant hoops to jump through. I am tired of Optometry Australia's toxic positivity. | | |
| 127 | | The lack of admin breaks, the strict nature of applying for annual leave 6 months in advance and not hearing any response for months, and the fact we have to work weekends unless we take a significant pay cut - all play a huge role in burn out in our company (Luxottica) | | |
| 128 | | The management structure lacks a practice manager who is present and aware of day-to-day activities of staff (admin and optometrist), as such, it makes it difficult to make suggestions for improvement to the business manager who is not present daily and understands staff dynamics. The business manager also lacks optometry background, so I am unsure whether they fully understand the profession and the role optometrists play in an ophthalmology practice. In most cases, focus is predominantly on running the business and ensuring the business is making profit. There is limited focus on professional development, and rewarding staff for their hard work. Management also lacks the ability to manage a team, instead picks on individual staff, over works them which often results in resignations and high staff turnover. Reality is we have a great time, however, management puts limited effort in understanding individual strengths and weaknesses to help staff work as a team to run the business. | | |
| 129 | | The only people winning in the corpeate setting is the HR staff, managers and Corperate Staff.  While optometrist and Patinets are loosing.  Contratuations OA on this accopishment.  Regards | | |
| 130 | | The oversupply in metro areas means that as an optometrist you are locked in to the practice of which you are currently employed. I was previously a partner at a specsavers store for 10 years and left due to growing dissatisfaction and issues regarding how corporate optometry was diminishing the value of patient care and has no regard for optometrist well being. I am now stuck in an independent practice where the owner has no respect for optometry and staff. | | |
| 131 | | The real issue is oversupplying of optometrist by creating more university places for optometry. We are walking the path of Pharmacy. | | |
| 132 | | The state of optometry in Australia, specifically Victoria is dire. There is a massive oversupply of optometrists which makes it impossible for us to negotiate better salary and working conditions. Voicing any dissatisfaction is an invitation for dismissal as we are easily replaced. Opportunities to change jobs are scarce too so we end up stuck in our current roles while mental health continues to deteriorate. I feel sorry for all young optometrists having to face these increasingly difficult and unfair working conditions without any indication that things will improve. | | |
| 133 | | The whole profession is an absolute joke. Why stay within an industry that values your sales skills more than your ability to provide health care? Why stay in a stagnating industry (salary growth) when other industries are making real changes to keep up with wage? Why stay in an industry that will burn you out like used incense and happily replace you with cheap graduates? No, I don't think this is a good profession to work in. It is not good financially, it is not good for your mental health. I would not recommend this career for anybody and would warn any students to consider pivoting to a different career. | | |
| 134 | | There are far too many optometrists in Australia, and this the root cause of the problem which you are trying to find out, but unfortunately in your survey there was not even one question to ask the participants about it. When there are too many of us, of course no one values us!. Those, who established school of Optometry in Flinders, Deakin, Canberra and Perth universities were a bunch of selfish pricks and traitors to the Optometry community in this country. The damage has been done, Big boys in the market(specsavers, Luxotica and the others) won spectacularly, thanks to inept leadership of the Optometrist association in South Australia, Victoria and at the national levels, but not in New South Wales or Queensland. The leader ships in the last 2 states were loyal to their members and were too strong and did not budge to the bulling tactics of the big boys in the industry. | | |
| 135 | | There are too many KPIs and I feel like I am not valued at OPSM as an Optometrist, but rather as someone to drive KPIs, which I really hate. I've been looking for a new job online (not a corporate practice) for the last 2 years but barely any jobs get advertised. | | |
| 136 | | There are too many universities and optometry grads flooding the market driving down wages and job opportunities!! | | |
| 137 | | They only care about patient numbers and profits, not your well-being. They act like they care, say that they're open to input, but would brush them off after you've voiced your concerns and turn around and place the blame on you instead. | | |
| 138 | | This is a follow up survey after an initially submitted survey (where I indicated I am currently full time employed at an independent practice but had recently been primarily working as a locum for several Specsavers stores). | | |
| 139 | | This survey is overdue and my story is only one of many that I hope you are able to hear.  As an early career optometrist, I have found the past 3 years eye-opening and disheartening. I'm now at a point where I'm seriously reconsidering my future career options. It is disappointing to study hard for years only to end up in a corporate practice/OPSM who treats you like an overpaid retail assistant and focuses more on your conversion than actual patient care. The industry is over-saturated in major cities, creating less job opportunities to move from these toxic work environments. This also allows the large corporations to add extra pressure as they know 'we are trapped' and if we choose to leave, they'll just "replace with a cheaper Graduate Optometrist".   The following things have happened to me and/or colleagues;  - Management has banned all admin slots. If they see any they remove them without your consent. Ie. urgent referral to hospital or end of day to finalise your work. You are told "Admin slots take up valuable conversion slots".  - Management refusing to pay overtime when Optometrists stay after hours to complete referrals/notes. This is compounded by the fact our rostered hours align with store opening hours. Ie. you are booked out from 8.30am to 5.30-6pm. You are told "it's your fault for not having better time management". - As a result of NO admin slots, its become normal for Optometrists to come into clinic on their days off/unpaid to complete referrals to meet medico-legal requirements. (This leads to further burn out because they are not taking the time to rest at home).   - Its normal to not take a full 30 minute lunch break or quick toilet break due to full appointment books. Sometimes, we skip lunch altogether and "forget to eat" to meet the demand of busy stores. (This is extremely unhealthy and leads to higher rates of burn out).  - No refusal of walk-ins even if you are fully booked. Store managers are told to add the patients in without consent and then management questions why you run over-time.  - Promoting "no health or follow up appointments" during sale weeks to avoid "time-wasters" and boost sales.  - Not approving annual leave days; even with requests 6 months in advance. Ie. My colleague wanted just 1 day off for her birthday but was told no its a sale day.   - Consistently told your conversion rate is "too low &lt;40%" and strongly encouraged to "avoid booking in the health only/emergency patients" ie. red eyes & flashes.   - Added stress of Management sitting in on your appointments every month and then criticising every word and process you did. (This could be a helpful tool but one manager takes it too far).  - You must provide a "valid reason" why each patient you saw didn't convert or buy products and email it to your manager daily.  - Optometrists refusing to see red eye patients. Reasoning being "it will only bring down my conversion rates." (This leads to unfair competition between practitioners and some are left with more of the clinical work and low performances.)  - Pressure from management to not schedule follow-up appointments, as this "takes up valuable conversion slots" ie. reviews of uveitis/red eyes. (This is dangerous and I have several examples of patients I had to re-treat as they developed secondary conditions from poor follow up by other colleagues).  - Manipulation and bullying tactics used to scare optometrists into working any roster the company wants, especially every Saturday and Sunday. If you want to have better life balance and not follow this weekend roster, you lose $20,000 from your salary. They give you no opportunity to negotiate or discuss, even though you are still working the same hours. | | |
| 140 | | This was a challenging questionnaire to do as a locum as from practice to practice it varies a lot. I know there is a lot of uncertainty in the air at the moment with people worried supply of Optometrist will over run demand. | | |
| 141 | | Time pressure, lack of allocated admin time are the main stressors. | | |
| 142 | | Too many optometry students graduating  No jobs available. Need to reduce the number tof optometry graduates each year.   Optometrists should advocate for Medicare to do penalty bulk billing rates on weekends and public holidays just like gps  Corporates need to raise salaries of optometrists | | |
| 143 | | Unfortunately the field of Optometry in Australia is dying. When colleagues who have studied 7 years cannot find work unless it's in rural towns only this can lead to many friends having depression. I have had to move to a different career and upskill there as well as being an optometrist just because the field of optometry is not looking bright for the majority. Yes, private practice settings allow for more freedom, to practise freely but that is less than 50% of optometry and the majority work in corporates. In the U.S, optometrists have the ability to upskill and reduce the burden on the healthcare system by doing procedures such as PLI, YAG laser capsulotomies, prescribe oral medication, even LASIK. RANZCO is the biggest hurdle facing optometrists. They act like colleagues and want to be our friends, but they are the first to say no to any upskilling optometrists want to do. When optometry colleagues who have studied 7 years now work as orthoptists in ophthalmology clinics for half the hourly rate they used to get two years ago, it says a lot about the future of the field. When 300 new graduates come out every year having to locum in their first year because they can't find jobs without moving to the other side of the country it says a lot about the future of the field. The universities are full of academics who have no clue about optometry out there. Many do great work in research and contribute greatly to clinical practice. But for what? we cannot even do half the things they teach us at university. Universities are the biggest frauds in the future of optometry. As long as they're making the money per student with the state governments getting a cut, they have no desire in inspiring the next future of health professionals nor care for their future in the field once leaving the university. When OA does a seminar promoting optometrists to move to different fields or get a second job that says a lot about the future. At least the U.S had the guts to lobby against the big corporates to moving in. The future is not bright at all. | | |
| 144 | | Unfortunately, business imperatives often override any feedback provided by staff. Staff are reluctant to voice opinions that may go against the plans of higher management, for fear of being perceived as negative. Micromanagement and favoritism are an issue. Optometrist redundancies in one major chain in recent times have created anxiety amongst staff. Lack of Medicare funding and erosion of fees has resulted in optometrists being underpaid for their time and expertise. Practices using high volume bulk billing models have not helped optometry's cause , often diminishing patient confidence and trust in practitioners. As a practitioner of 30 years standing, I am concerned about the future of the profession, it's viability and sustainability. Already we are seeing an oversupply in metro Melbourne, which gives employers the upper hand as far as them adopting the 'take it or leave it approach ' , with wages far below the worth of the practitioner. | | |
| 145 | | Very grateful to at least be working in independent optometry, but as I locum an extra day around once per month at various places, I can see such a difference in the level of care (lower) at other places especially where testing is only 20minutes (often including pretesting) despite us getting the same amount of rebate back from Medicare. I think there should also be a minimum standard of equipment in each practice, some places (Oscar Wylee) don’t even offer contact lenses and up until recently did not have visual field or fundus cameras so other practices had to pick up their slack in terms of eye health care, which would also be costing the government more money in Medicare fees. I have had friends and family comment on how different their eye tests have been at different practices, locations etc, and I think bare minimum standards for 10916/10918 or 10910 consultations need to be made clearer and adhered to. (Examples of this: some friends have said they had no ocular health checked, or no slit lamp used, prescription check being 2 mins i.e. vert their specs, put in trial frame, see they can read 6/6 and then copy the same prescription from before) | | |
| 146 | | Waiting times and workload varies depend on time of the year. Waiting times for my patients specifically to see me is also longer due to the nature of my work hours. My overall job satisfaction as a corporate employed optometrist is low, although I do feel professionally valued within my practice (store), I do not feel the same value in the larger environment (i.e. within the company or the industry). I do not wish to stay in this profession in 5 years' time. | | |
| 147 | | We are given no autonomy. We are not respected. We make no decisions. We have to compromise on care. Optometry has been ruined by corporates and the previous generation who sold out to them. Most people want to quit. We feel betrayed that we by the universities that knew full well they were pushing out to many graduates (there is plenty of data reinforcing this before even more schools were oponed). | | |
| 148 | | we should be paid more for what we do - from Medicare but also our salaries from employers | | |
| 149 | | We’re in the process of moving from an independent to a corporate practice, which is why some of my answers are mixed. I had a wonderful workplace that is rapidly becoming a workplace we are all actively trying to leave. | | |
| 150 | | What you you are not capturing in the questions you are asking is the relentlessness of working at Specsavers literally one after another and going home feeling brain dead  What you are not capturing is the KPI pressure at Luxottica and the pressure to upsell an optos to everyone for $60. Then the dreaded Tuesday night email where your Optos upsells are ranked in a league table and the laggards are berated   What you are also not capturing is the collapse of the locum market in WA following the opening of the UWA course. Think job insecurity   Also no mention of the fate of the new grads. It is cruel what is going to be happening to them. The market in Perth is now absolutely saturated. Here is the problem if you don’t get a job right after graduation you will lose all your optometry skills. A grad couldn’t spend a year doing hospitality and jump back into optometry. Since it is such a specialized level of training there are not many opportunities elsewhere compared to other degrees like law or business. If a new grad doesn’t get a job right away it’s basically 3 years and $150000 down the toilet | | |
| 151 | | Who is ultimately responsible for the well being of the staff at work?  Are University accountable ethical, moral and legal liable for the students, current optometry cohort and the wider community? Are big conpanies responsible for sustaining the profession? | | |
| 152 | | Why is the qld oaa making it more difficult and expensive to achieve required cpd points as the do not run any conference | | |
| 153 | | Working in a large corporation, you don't get any say as to how things are run.   The introduction of Clarifye has not been welcomed by most optometrists here, it is totally unnecessary and not even that accurate. Yet a lot of money was spent on it by the company, I'm not sure who they consulted when they decided to roll that out.   They have also brought in new visual field machines and OCT equipment, however they have not backed up existing data. This is very problematic to me and I am annoyed by this. | | |
| 154 | | Working on 20 minute appointments and no longer able to put in any breaks if running behind, referrals or reports to write, etc. If we extend an appt to 40min because of language barriers, special needs, age, etc all this has to be clearly documented in the appointment book. Regional and national managers watch appointment books and will delete appintment placeholders to the stage that if we need a break, we’ll just make up a patient that will “no show”. Our company has a rule/ideal of no more than 60min of “clinical” appts per day (ie, not converting appts - in this includes children, CL F+T, VF, CL aftercare, red eyes) Our company has been pushing for us to do more contact lenses (the KPI of 5 new fits per week) but give us no time to do this and we get harassed if we are seeing too many contact lens patients. | | |
| 155 | | working with dispensers that have zero knowledge and no motivation for optometry makes my job harder and harder. They arent willing to learn and the company invest zero money in training them. This makes me hate my job | | |
